# Distinct Opsin 3 (*Opn3*) Expression in the Developing Nervous System during Mammalian Embryogenesis

**DOI:** 10.1523/ENEURO.0141-21.2021

**Published:** 2021-09-09

**Authors:** Wayne I. L. Davies, Soufien Sghari, Brian A. Upton, Christoffer Nord, Max Hahn, Ulf Ahlgren, Richard A. Lang, Lena Gunhaga

**Affiliations:** 1 Umeå Centre for Molecular Medicine (UCMM), Umeå University, Umeå 901 87, Sweden; 2 Medical Scientist Training Program, University of Cincinnati, Cincinnati, OH 45229; 3 The Visual Systems Group, Abrahamson Pediatric Eye Institute, Cincinnati Children’s Hospital Medical Center, Cincinnati, OH 45229; 4 Center for Chronobiology, Division of Pediatric Ophthalmology, Cincinnati Children’s Hospital Medical Center, Cincinnati, OH 45229; 5 Division of Developmental Biology, Cincinnati Children’s Hospital Medical Center, Cincinnati, OH 45229; 6 Department of Ophthalmology, University of Cincinnati, College of Medicine, Cincinnati, OH 45221

**Keywords:** brain, development, encephalopsin, nervous system, Opn3, OPT

## Abstract

Opsin 3 (*Opn3*) is highly expressed in the adult brain, however, information for spatial and temporal expression patterns during embryogenesis is significantly lacking. Here, an *Opn3*-eGFP reporter mouse line was used to monitor cell body expression and axonal projections during embryonic and early postnatal to adult stages. By applying 2D and 3D fluorescence imaging techniques, we have identified the onset of Opn3 expression, which predominantly occurred during embryonic stages, in various structures during brain/head development. In addition, this study defines over twenty *Opn3*-eGFP-positive neural structures never reported before. *Opn3*-eGFP was first observed at E9.5 in neural regions, including the ganglia that will ultimately form the trigeminal, facial and vestibulocochlear cranial nerves (CNs). As development proceeds, expanded *Opn3*-eGFP expression coincided with the formation and maturation of critical components of the central and peripheral nervous systems (CNS, PNS), including various motor-sensory tracts, such as the dorsal column-medial lemniscus (DCML) sensory tract, and olfactory, acoustic, and optic tracts. The widespread, yet distinct, detection of *Opn3*-eGFP already at early embryonic stages suggests that Opn3 might play important functional roles in the developing brain and spinal cord to regulate multiple motor and sensory circuitry systems, including proprioception, nociception, ocular movement, and olfaction, as well as memory, mood, and emotion. This study presents a crucial blueprint from which to investigate autonomic and cognitive opsin-dependent neural development and resultant behaviors under physiological and pathophysiological conditions.

## Visual Abstract

**Figure:**
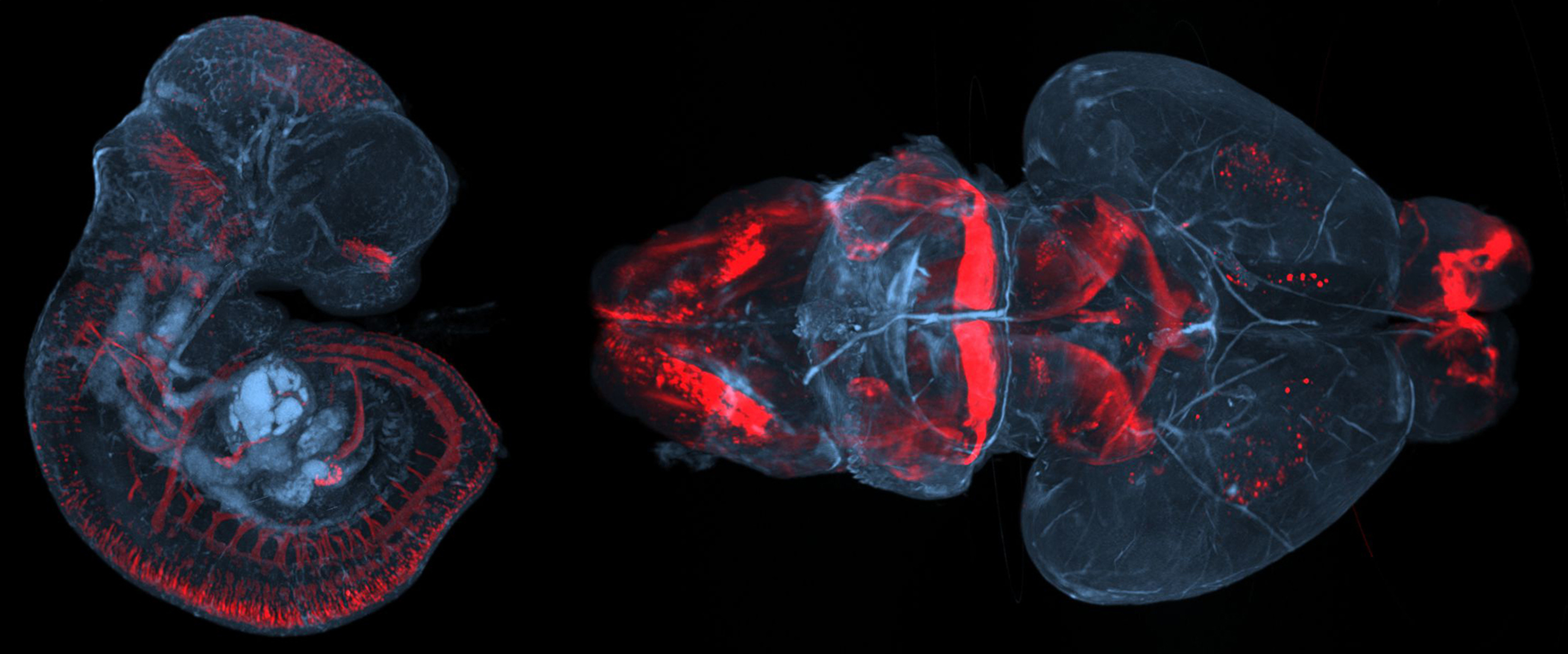

## Significance Statement

The expression of the mammalian Opsin 3 (*Opn3*) has only recently been characterized in adults, with no significant study during embryonic development. This study used an *Opn3*-eGFP mouse line to identify *Opn3*-related development of the central and peripheral nervous systems (CNS, PNS). 2D and 3D fluorescence imaging revealed cell body and axonal *Opn3*-eGFP expression, which indicated an early onset of *Opn3*-eGFP in cranial and spinal nerve ganglia, and sensory organs. Embryonic expression of *Opn3*-eGFP was also identified in the brainstem, cerebellum, hypothalamus and thalamus, whereas *Opn3*-eGFP expression in the cerebral cortex and associated limbic system regions were observed postnatally. The presence of Opn3, a short-wavelength-sensitive photopigment, in many brain regions during embryogenesis is of great interest when investigating autonomic and cognitive photo-dependent neural development.

## Introduction

Light detection (photoreception) and its related disorders are often focused on the visual system, where eyes and the specialized rod and cone photoreceptors they contain are crucial for forming images of the external world. However, it has become evident that there is a multiplicity of non-visual light detection processes, where specialized G-protein-coupled receptors (i.e., opsins) mediate both visual and non-visual photoreception. Briefly, the opsin protein moiety is covalently linked to a light-sensitive retinoid chromophore, which together form a photopigment (Yokoyama, 2000; Davies et al., 2012). In the presence of particular wavelengths of light, photopigments absorb the energy of a photon and, thereafter, activate functionally relevant phototransduction signaling cascades (Shichida and Matsuyama, 2009; Lamb et al., 2016).

For many years, it was assumed that visual opsins in the rods and cones, and a subset of intrinsically photosensitive retinal ganglion cells (RGCs; see extended data for a list of abbreviations) were the only opsin-based photoreceptors present in mammals, all of which were restricted to the retina. However, ∼20 years ago, so-called atypical or non-visual opsins, including Opsin 3 (*Opn3*, also known as encephalopsin or panopsin), *Opn4* (melanopsin), and *Opn5* (neuropsin), were identified in photoreceptors that were not thought to be involved in visual perception directly (Provencio et al., 1998; Blackshaw and Snyder, 1999; Tarttelin et al., 2003b). Of these three opsins, melanopsin has been the most studied in terms of function in visual and non-visual light detection processes, where it has been shown to play a key role in circadian clock photoentrainment and pupillary constriction in mammals (Freedman et al., 1999; Lucas et al., 2003; Xue et al., 2011; Sghari et al., 2020). Other known non-visual effects of *Opn4* include DNA repair activation (Hirayama et al., 2009) and cell cycle regulation (Dekens et al., 2003). Nevertheless, in mammals, relatively little is known about non-visual light detection processes.

Consistent with reported light-regulated processes other than vision, the presence of atypical photopigments in multiple non-retinal adult tissues has been observed. For example, *Opn4* has been detected in melanocytes (Provencio et al., 1998), the iris (Xue et al., 2011; Sghari et al., 2020), cornea and associated trigeminal ganglia (Delwig et al., 2018), blood vessels and heart (Regard et al., 2008; Sikka et al., 2014), and *Opn5* being found in the eye, brain, testes, ear, and skin (Kojima et al., 2011; Buhr et al., 2015, 2019; Haltaufderhyde et al., 2015). The initial identification and expression of *Opn3* in the adult mouse brain, including regions like the cerebral cortex and cerebellum (Blackshaw and Snyder, 1999), have been verified and extended (Nissilä et al., 2012; Olinski et al., 2020b). Recent studies in the adult mammalian brain, especially that of rodents and some primates, indicate that *Opn3* exhibits a far wider expression profile (Nissilä et al., 2012; Olinski et al., 2020b), which suggests the potential for many unknown roles of light signaling in the brain and other organs. Notably in adult vertebrates, including higher mammals, *Opn3* is expressed in adipocytes and several tissues, such as, heart, intestine, kidneys, liver, ovaries, pancreas, placenta, pulmonary arteries, retina and associated eye structures, skeletal muscle, skin, testes, uterus, trachea, and lungs (Blackshaw and Snyder, 1999; Halford et al., 2001; Tarttelin et al., 2003a; Haltaufderhyde et al., 2015; Ortiz et al., 2018; Nayak et al., 2020; Olinski et al., 2020a; Yim et al., 2020; Wu et al., 2021). In addition, phylogenetic analyzes indicate that related orthologues of *Opn3* are present in all major vertebrate classes studied to date (Shichida and Matsuyama, 2009; Davies et al., 2015), suggesting a conserved function for this photopigment. The widespread adult expression pattern of these non-visual opsins suggests that light may play a broader functional role than previously thought. Whether non-visual opsins are also spatially and temporally expressed during embryonic stages, and if so, in which structures and when (i.e., the time of onset), have not been defined.

A sufficient number of photons is able to penetrate the skin, skull, soft tissues, including the mammalian brain itself, at both fetal and adult stages to activate photoreceptors (Dunn et al., 2015; Sun et al., 2016; Reid et al., 2017; Nayak et al., 2020; Zhang et al., 2020). Therefore, it is essential to determine whether Opn3 might play a significant role in brain development and function. As such, the primary aims of this study were to use an *Opn3*-eGFP reporter mouse model to investigate the expression of *Opn3* throughout embryonic development, just after birth, as well as juvenile and adult stages. A combination of immunofluorescence techniques, including 3D imaging by optical projection tomography (OPT) was used.

This study provides a detailed appreciation of the early onset of *Opn3* at around embryonic day (E)9.5 and broad, yet distinct, expression of *Opn3* in the developing mammalian embryo. Specifically, numerous *Opn3-*eGFP*-*positive cranial ganglia, neural projections and nuclei were detected in the developing brain and spinal cord, which together identify key structures of major neural pathways and circuits. Collectively, these results form the basis of further investigations into how Opn3-dependent signaling might regulate the development of the nervous system and other key structures, which might prove significant in understanding the season-of-conception/birth dependent risk of developing several metabolic, neurologic and psychiatric diseases (Foster and Roenneberg, 2008).

## Materials and Methods

### Ethics statement

All animal procedures were performed in accordance with the Umeå University animal care committee’s regulations.

### *Opn3-*eGFP reporter mice

A genetically-modified *Opn3*-eGFP (enhanced green fluorescent protein) reporter mouse strain (*Tg[Opn3-EGFP]JY3Gsat*; MMRRC/GENSAT stock number 030727-UCD, Mutant Mouse Resource and Research Centers, University of California, Davis, CA) was generated as detailed previously (Nayak et al., 2020). This reporter mouse strain contains an *eGFP* reporter gene inserted at the start of exon 1 of the coding sequence of *Opn3*, but downstream of the *Opn3* promoter. As such, eGFP expression was driven by the *Opn3* promoter and is stated as *Opn3*-eGFP herein. Animals were housed on a 12/12 h light/dark (LD) cycle in a temperature and humidity-controlled environment.

### Genotyping and PCR

Genotyping to confirm the presence of the *Opn3*-eGFP allele was performed using an oligonucleotide pair (forward primer, 5´-CAGAGCGTGAGATCCACCCTGTT-3´; reverse primer, 5´-TAGCGGCTGAAGCACTGCA-3´) that generated an amplicon of 320 bp under the following PCR cycling conditions: an initial step of 94°C for 5 min, followed by 30 cycles of 94°C for 30 s, 56°C for 30 s, and 72°C for 30 s, and a final elongation step of 72°C for 7 min.

### Tissue preparation

Adult mice and pregnant females (one to five months) were perfused with fresh 4% paraformaldehyde (PFA) in 0.1 m phosphate buffer (PB; pH 7.4) that was filtered through a 0.2 μm syringe filter to remove particulates. Where stated, whole embryos, heads or brains from embryonic stages (E8.5, E9.5, E10.5, E13.5, E15.5, and E17.5), as well as from postnatal (P) stages 0.5, P10, and adults, were collected. At least three and up to five specimens of each time point were analyzed. In all cases, retrieved embryonic, postnatal or adult tissues were immersion-fixed in fresh filtered PFA (4%) at 4°C for 4 h to overnight (ON), before being stored in fresh 0.1 m PB at 4°C until further use.

### Immunohistochemistry

Gradual cryo-protection of whole embryos or selected tissues using 3.25–25% sucrose in PB at 4°C was performed, and thereafter embedded in NEG-50 (Cellab). Samples were stored at −80°C and cryo-sectioned with thickness at 20 μm (for E8.5–E10.5), 40 μm (for E13.5 to adult stages), or 10 μm (for E17.5 *Opn3*-eGFP/GFAP staining). For *Opn3*-eGFP reporter detection, immunohistochemistry was used by applying standard protocols (Wittmann et al., 2014) with a rabbit anti-GFP antibody (1:500 dilution; Thermo Fisher Scientific; #A11122) and an anti-rabbit IgG Cy3-conjugated secondary antibody (1:400 dilution; Jackson ImmunoResearch Europe; #111-165-003), or an anti-rabbit Alexa Fluor 488-conjugated secondary antibody (1:300, Jackson ImmunoResearch). For glial fibrillary acidic protein (GFAP) and *Opn3*-eGFP co-labeling, rabbit anti-GFAP (1:500 dilution; gift from Anna Överby Wernstedt, Umeå University) and goat anti-GFP (1:500 dilution; Thermo Fisher Scientific; #A600-101-215M) primary antibodies were used. Secondary antibodies used were; donkey anti-rabbit Alexa Fluor 594-conjugated (1:400 dilution; Thermo Fisher Scientific; #A21207) and donkey anti-goat Alexa Fluor 488-conjugated antibodies (1:400 dilution; Thermo Fisher Scientific; #A11055; gift from Helena Edlund, Umeå University), respectively. In all cases, 4′,6-diamidino-2-phenylindole (DAPI; 1:400 dilution; Sigma-Aldrich) was included to counterstain nuclei. Slides were mounted with fluorescent mounting medium (Agilent Technologies). Immunohistochemistry sections were examined by fluorescence microscopy (Nikon Eclipse, E800; Nikon Instruments Europe), with images being captured using a Nikon DS-Ri1 digital camera and Nikon NIS-Elements F v4.6 software, before being processed with Photoshop Creative Cloud (CC) 2019 (Adobe).

### OPT

OPT scanning was performed on whole embryos at E10.5 and brains at P0.5. Sample preparation was performed essentially as previously described (Sharpe et al., 2002; Alanentalo et al., 2007; Eriksson et al., 2013), except for the addition of further freeze-thaw methanol steps to increase antibody permeabilization (for both samples at E10.5 and P0.5) and extended washing steps for P0.5 brain samples. For *Opn3*-eGFP detection, filtered (0.45 μm) rabbit anti-GFP (1:500 dilution, Thermo Fisher Scientific; #A11122) and goat anti-rabbit IgG Cy3-conjugated secondary (1:400 dilution; Jackson ImmunoResearch Europe; #111-165-003) antibodies were used, incubating at RT for 48–72 h at each step. Specimens were briefly equilibrated and placed in filtered low melting point agarose (1.5%; SeaPlaque Agarose, Lonza) and mounted into positioned, before methanol treatment and clearing in benzyl alcohol and benzyl benzoate (ratio 1:2). Once adequately transparent, specimens were imaged using a Bioptonics 3001 OPT scanner (SkyScan) using Texas Red (Ex/Em: 560 ± 20 nm/610 nm LP; exposure time: 800 ms for E10.5 and 425 ms for P0.5) and FITC (Ex/Em: 425 ± 20 nm/475 nm LP; exposure time: 450 ms for E10.5 and 200 ms for P0.5) filter sets to maximize *Opn3*-eGFP and anatomic (autofluorescence) signals, respectively. Computational postscanning processing were conducted as previously described (Cheddad et al., 2012). 3D tomographic reconstructions were performed using NRecon v1.6.9.18 software (SkyScan), with visualization and video production conducted by implementing Imaris 9.3.1 software (Bitplane).

## Results

In this study, the spatial and temporal expression of Opsin 3 *(Opn3)* was analyzed by examining an *Opn3* promoter-driven eGFP mouse line (*Opn3*-eGFP) with a main focus on embryonic central and peripheral nervous systems (CNS, PNS). The detection of *Opn3*-eGFP expression was optimized, by using a sequential combination of a primary GFP and a secondary Cy3 antibody, to amplify positive staining and reduce unspecific background labeling. To define both temporal and spatial expression of *Opn3-*eGFP, embryos and postnatal animals of several stages were analyzed using sections in coronal, transversal and sagittal orientations. The below presented *Opn3-*eGFP expression patterns were consistent observations comprised from analyzing a minimum of three and up to five specimens of each time point.

### Early onset of *Opn3* expression in the developing nervous system

*Opn3-*eGFP expression was detected in the developing CNS and PNS at E9.5 (Extended Data Fig. 1-1), but not at E8.5 (data not shown). Cranial nerves (CNs) are part of the PNS that have both sensory and/or motor components that direct multiple sensory, motor and autonomic functions. The sensory part, including the ganglia, derives from cranial neural crest cells and ectodermal placode cells (Patthey and Gunhaga, 2011; Schlosser, 2014; Vermeiren et al., 2020). At E9.5, *Opn3-*eGFP expression was detected in cells of the early forming trigeminal ganglia and facio-acoustic ganglion complex, which will later give rise to the sensory branches of the trigeminal (CN V), facial (CN VII), and vestibulocochlear (CN VIII) CNs (Extended Data Fig. 1-1*B*,*C*,*J*,*K*). Furthermore, *Opn3-*eGFP-positive (*Opn3-*eGFP^+^) cells were observed in the basal plate of the neural tube along the anteroposterior axis of the future spinal cord (Extended Data Fig. 1-1*A–D*,*L*), as well as the second, third and fourth pharyngeal pouches (Extended Data Fig. 1-1*A–C*). Additional *Opn3-*eGFP^+^ non-neural regions, which are not the subject of this study, included the gastrointestinal tract, the dorsal pancreatic bud and the developing heart (Extended Data Fig. 1-1*A*,*B*,*D*).

Scattered *Opn3-*eGFP expression was also observed in the olfactory placode (Extended Data Fig. 1-1*E*,*F*), the most ventral and dorsal regions of the diencephalic neuroepithelium (Extended Data Fig. 1-1*G*,*H*) and the dorsal outer lining of the mesencephalic neuroepithelium (Extended Data Fig. 1-1*I*). Thus, the onset of *Opn3* expression is ∼E9.5 in both the CNS and PNS.

### At E10.5, *Opn3*-eGFP is expressed in several motor-sensory related cells

**Figure 1.**
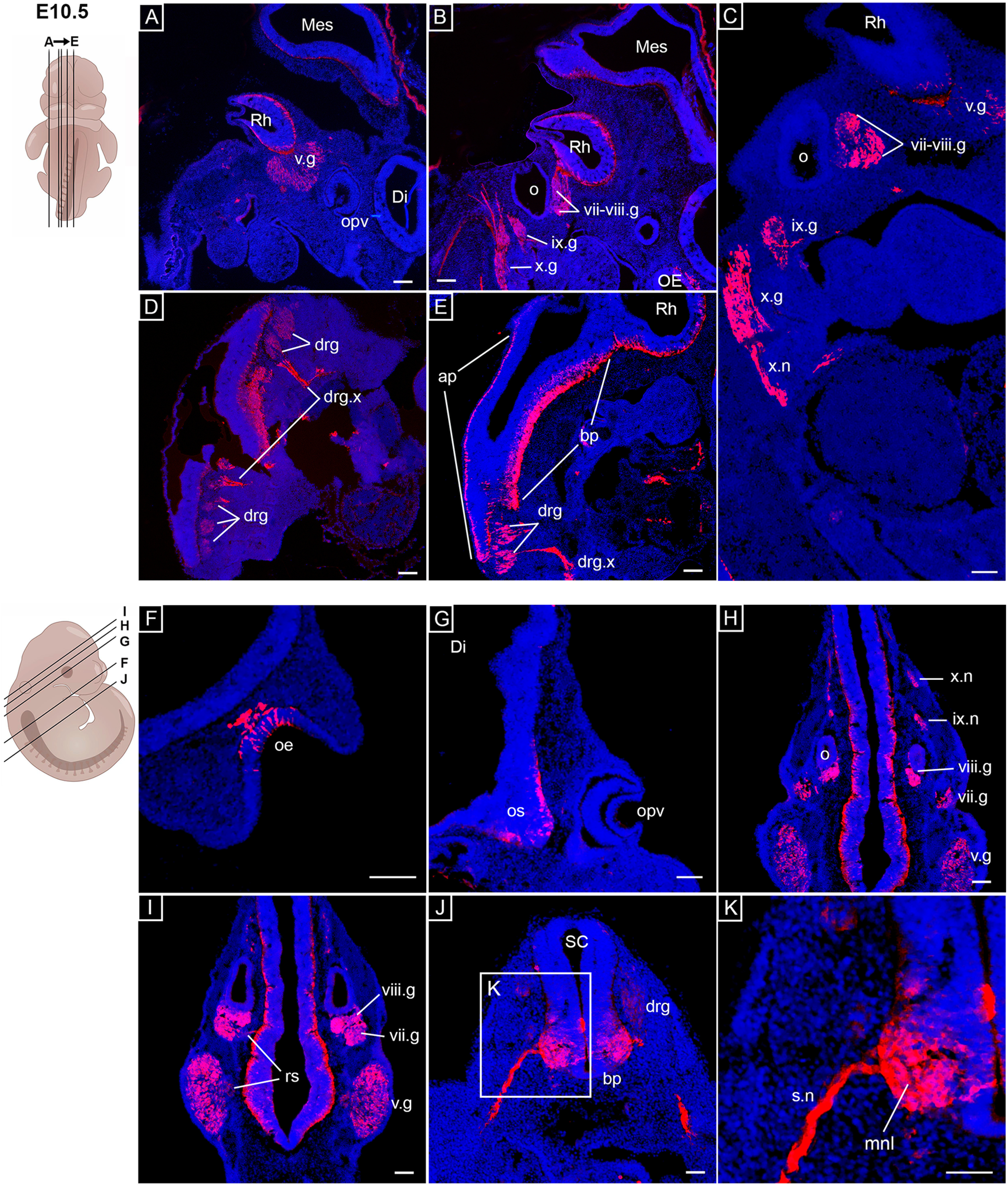
*Opn3*-eGFP immunodetection (red) at E10.5 in sagittal (***A–E***) and coronal (***F–K***) serial sections counterstained with DAPI (blue). On the left, schematics of E10.5 embryos including planes of sections shown in ***A–K***. ***A***, Di: diencephalic vesicle, Mes: mesencephalic vesicle, opv: optic vesicle, Rh: rhombencephalic vesicle, v.g: trigeminal ganglion. ***B***, ix.g: glossopharyngeal ganglion, Mes: mesencephalic vesicle, o: otic vesicle, oe: olfactory epithelium, Rh: rhombencephalic vesicle, vii-viii.g: facio-acoustic ganglia, x.g: vagus ganglion. ***A***, ***B***, Parts of the amnion surrounding the embryo were digitally removed (raw data in Extended Data Fig. 1-3). ***C***, ix.g: glossopharyngeal ganglion, o: otic vesicle, Rh: rhombencephalic vesicle, v.g: trigeminal ganglion, vii-viii.g: facio-acoustic ganglia, x.g: vagus ganglion, x.n: vagal nerve. ***D***, drg: dorsal root ganglia, drg.x: dorsal root afferent processes. ***E***, ap: alar plate, bp: basal plate, drg: dorsal root ganglia, drg.x: dorsal root afferent processes, Rh: rhombencephalic vesicle. ***F***, oe: olfactory epithelium. ***G***, Di: diencephalic vesicle, opv: optic vesicle, os: optic stalk. ***H***, ix.n: glossopharyngeal nerve, o: otic vesicle, v.g: trigeminal ganglion, vii.g: geniculate ganglion, viii.g: vestibulocochlear ganglion, x.n: vagal nerve. ***I***, rs: rootlets of the v/vii/viii.g, v.g: trigeminal ganglion, vii.g: geniculate ganglion, viii.g: vestibulocochlear ganglion. ***J***, bp: basal plate, drg: dorsal root ganglion, SC: spinal cord. ***K***, mnl: mantle layer, s.n: spinal nerves. Scale bars: 100 μm. Extended Data Figures 1-1, 1-2 are supporting this figure.

At E10.5, *Opn3-*eGFP expression was detected in the expanding trigeminal ganglia near to the brainstem (Fig. 1*A*,*C*,*H*,*I*), in the facio-acoustic ganglion complex in close connection to the *Opn3-*eGFP negative (*Opn3-*eGFP*^-^*) otic vesicle (Fig. 1*B*,*C*,*H*,*I*), in the developing glossopharyngeal and inferior vagal ganglia, including peripheral projections of the vagal ganglia (Fig. 1*B*,*C*,*H*). Scattered *Opn3-*eGFP^+^ cells were observed in the neuroepithelium of the brainstem at the level of the trigeminal ganglia, in contrast to the level of the facio-acoustic complex and otic vesicle, where *Opn3-*eGFP^+^ cells were primarily located in the lateral lining of the brainstem neuroepithelium (Fig. 1*H*,*I*). Moreover, *Opn3-*eGFP was detected in the dorsal root ganglia, derived from neural crest cells, throughout the anteroposterior axis of the spinal cord (Fig. 1*D*,*E*,*J*). Several afferent processes of the dorsal root ganglia were also observed in the hindbrain region (Fig. 1*D*,*E*). Consistent with findings at E9.5 (Extended Data Fig. 1-1*H–K*), *Opn3-*eGFP^+^ cells were observed along the basal plate of the caudal neural tube, including motor exit points and related peripheral spinal nerve projections (Fig. 1*J*,*K*; Extended Data Fig. 1-2*A–C*).

10.1523/ENEURO.0141-21.2021.f1-1 Extended Data Figure 1-1 *Opn3*-eGFP immunodetection (red) at E9.5 in sagittal (***A–D***) and coronal (***E–L***) serial sections counterstained with DAPI (blue). On the left, schematics of E9.5 embryos including planes of sections shown in ***A–L***. ***A***, bp: basal plate, dpb: dorsal pancreatic bud, nt: neural tube, php: pharyngeal pouches. ***B***, dpb: dorsal pancreatic bud, nt: neural tube, php: pharyngeal pouches, v.g: trigeminal ganglion. ***C***, bp: basal plate, nt: neural tube, o: otic vesicle, php: pharyngeal pouches, vii-viii.g: facio-acoustic ganglia. ***D***, git: gastrointestinal tract, Mes: mesencephalic vesicle, Pro: prosencephalic vesicle, Rh: rhombencephalic vesicle. ***E***, Te: telencephalic vesicle. ***F***, op: olfactory placode, Te: telencephalic vesicle. ***G***, Di: diencephalic vesicle, inf: infundibulum. ***H***, Di: diencephalic vesicle. ***I***, Mes: mesencephalic vesicle. ***J***, Mes: mesencephalic vesicle. ***K***, o: otic vesicle, Rh: rhombencephalic vesicle, v.g: trigeminal ganglion, vii-viii.g: facio-acoustic ganglia. ***L***, bp: basal plate, nt: neural tube. Scale bars: 100 μm. Download Figure 1-1, TIF file.

**Figure 2.**
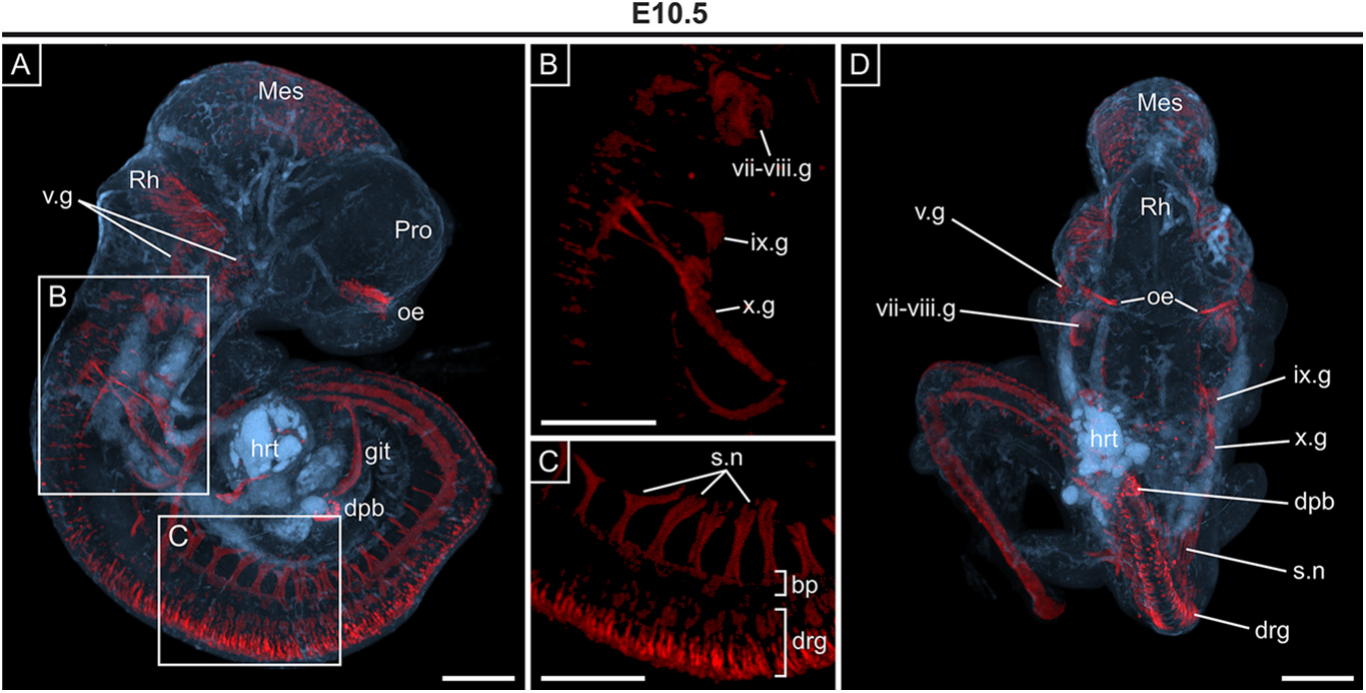
3D OPT imaging of whole E10.5 embryo showing *Opn3*-eGFP immunodetection. ***A***, Sagittal view showing detection of *Opn3*-eGFP (red) against a background of auto-fluorescing anatomical structures (blue). Developing ganglia that form the sensory branches of CNs V, CN VII, and CN VIII CNs are shown in ***B***, and spinal nerves highlighted in ***C***, ***D***. ***D***, Dorsal view of embryo shown in ***A***. bp: basal plate, dpb: dorsal pancreatic bud, drg: dorsal root ganglia, git: gastrointestinal tract, hrt: heart; ix.g: glossopharyngeal ganglion, Mes: mesencephalic vesicle, oe: olfactory epithelium, Pro: prosencephalic vesicle, Rh: rhombencephalic vesicle, s.n: spinal nerves, v.g: trigeminal ganglion, vii-viii.g: facio-acoustic ganglia (comprising the vii.g geniculate ganglion and viii.g: vestibulocochlear ganglion), x.g: vagus ganglion. Scale bars: 250 μm.

10.1523/ENEURO.0141-21.2021.f1-2 Extended Data Figure 1-2 No DAPI labelled nuclei were detected in *Opn3*-eGFP-positive projection areas. ***A–F***, *Opn3*-eGFP immunodetection (red) at E10.5 in (***A–C***) and at E13.5 (***D–F***) horizontal sections counterstained with DAPI (blue). ***A***, bp: basal plate, drg: dorsal root ganglion, SC: spinal cord. ***B***, s.n: spinal nerves, mnl: mantle layer. ***C***, s.n: spinal nerves. ***D***, dpcb: deep nuclei of cerebellum, Tec: tectum of midbrain, v.mes: mesencephalic trigeminal nucleus. ***E***, v.spn: spinal trigeminal nerve, vii: facial nerve, vii.gn: genu of facial nerve. ***F***, v.spn: spinal trigeminal nerve, vii: facial nerve, vii.gn: genu of facial nerve. Scale bars: 100 μm. Download Figure 1-2, TIF file.

**Figure 3.**
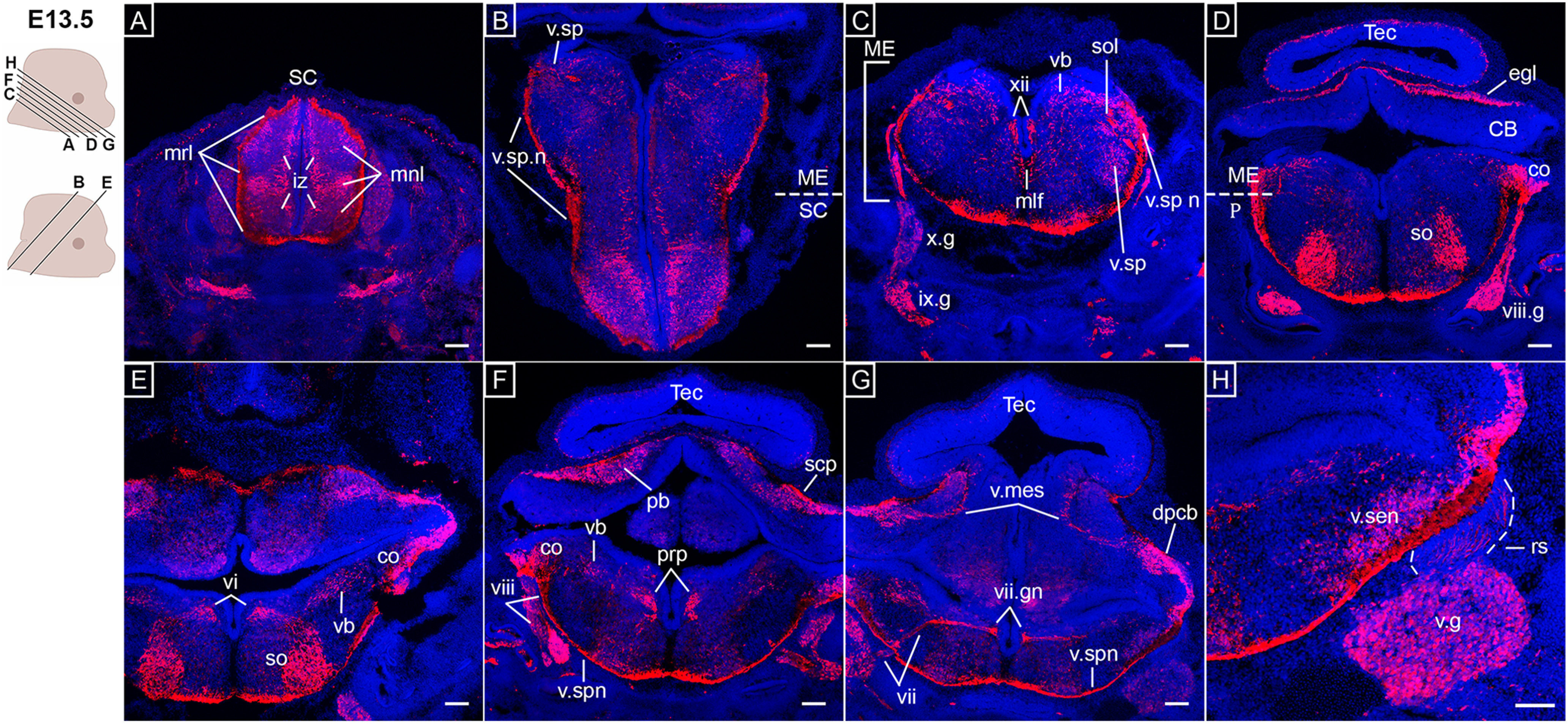
*Opn3*-eGFP immunodetection (red) at E13.5 in horizontal (***A***, ***C***, ***D***, ***F–H***) and coronal (***B***, ***E***) serial sections in caudo-rostral direction from the spinal cord to hindbrain areas counterstained with DAPI (blue). Left, Schematics of E13.5 embryonic heads including planes of sections shown in ***A–H***. ***A***, iz: intermediate zone, mnl: mantle layers, mrl: marginal layers, SC: spinal cord. ***B***, ME: medulla, SC: spinal cord, v.sp n: spinal trigeminal nerve, v.sp: spinal trigeminal nucleus. ***C***, ix.g: glossopharyngeal ganglion, ME: medulla, mlf: medial longitudinal fasciculus, sol: solitary tract, v.sp: spinal trigeminal nucleus, vb: vestibular nuclei, x.g: vagus ganglion, xii: hypoglossal nucleus. ***D***, CB: cerebellum, co: cochlear nucleus, egl: external granular layer of cerebellum, ME: medulla; P: pons, so: superior olivary complex, Tec: tectum of midbrain, vii.g: geniculate (facial) ganglion. ***E***, co: cochlear nucleus, so: superior olivary complex, vb: vestibular nuclei, vi: abducens nucleus. ***F***, pb: parabrachial nuclei, prp: nucleus prepositus, scp: superior cerebellar peduncle, Tec: tectum of midbrain, v.sp.n: spinal trigeminal nerve, viii: vestibular nerve. ***G***, dpcb: deep cerebellar nuclei, Tec: tectum of midbrain, v.mes: mesencephalic trigeminal nucleus, v.sp.n: spinal trigeminal nerve, vii.gn: genu of facial nerve, vii: sensory/parasympathetic facial nerve. ***H***, rs: rootlets of the v.g, v.g: trigeminal ganglion, v.sen: sensory trigeminal nucleus. Scale bars: 100 μm.

10.1523/ENEURO.0141-21.2021.f1-3 Extended Data Figure 1-3 Raw data of the main images in Figure 1*A*,*B*. *Opn3*-eGFP immunodetection (red) at E10.5 in horizontal serial sections counterstained with DAPI (blue) showing the (in Fig. 1*A*,*B*) digitally removed *Opn3*-eGFP-positive amnion surrounding the embryo (arrowheads). Scale bar: 100 μm. Download Figure 1-3, TIF file.

By E10.5, the thickened olfactory placode has invaginated: at this stage, *Opn3-*eGFP expression was observed in both the olfactory epithelium and the nearby mesenchyme (Fig. 1*F*), which resembled patterns of postmitotic olfactory neurons (Miller et al., 2010; Panaliappan et al., 2018). Expression of *Opn3-*eGFP was also detected in the developing optic stalk; but at this stage, no expression was observed in the optic cup (Fig. 1*G*).

To obtain a global appreciation of *Opn3-*eGFP expression, with particular reference to the identified projections, 3D imaging by OPT was conducted, which verified the aforementioned *Opn3-*eGFP expression pattern noted in E10.5 sections. This included detection of *Opn3-*eGFP in different components of the PNS, such as nerves, ganglia, as well as sensory (otic) and somatosensory (skin, thorax, abdomen and limbs) projections toward their CNS targets, the hindbrain via the rootlets of cranial ganglia and the spinal cord via the dorsal root ganglia, respectively (Fig. 2; Movie 1). The uniform upregulation of *Opn3-*eGFP in the dorsal root ganglia along the anteroposterior axis of the embryo, as well as in projections emanating from dorsal root and cranial ganglion, and the olfactory placode were clearly visible (Fig. 2; Movie 1). Consistent with expression observed in E9.5 sections (Fig. 1), *Opn3-*eGFP was also observed in abdominal structures and the developing heart (Fig. 2; Movie 1).

Movie 1. Video of 3D imaging of a whole E10.5 embryo (along the *y*-axis) showing *Opn3*-eGFP immunodetection (red) against a background of auto-fluorescing anatomical structures (blue). 10.1523/ENEURO.0141-21.2021.video.1

### *Opn3*-eGFP detection in distinct ganglia and nerve fibers at E13.5

At E13.5, *Opn3-*eGFP was detected in many regions of the CNS, including cells of both the mantle and marginal layers of the spinal cord (Fig. 3*A*). The visualization of *Opn3-*eGFP labeled spinal motor neurons at E10.5, before sensory neurons located in the mantle layer, are consistent with the chronological differentiation of spinal motor neurons that precedes sensory neurons (Rodier, 1980). At the level of the medulla, *Opn3-*eGFP was detected in the spinal trigeminal nucleus, which receives information about deep/crude touch, pain and temperature from the face via all three branches of CN V (trigeminal), in addition to branches of CN VII (facial), CN IX (glossopharyngeal), and CN X (vagus; Fig. 3*B*,*C*). Consistently, *Opn3-*eGFP detection was also noted in trigeminal ganglia rootlets projecting to the principal sensory trigeminal nucleus (Fig. 3*H*), which receive information about discriminative sensation and light touch of the face, as well as conscious proprioception of the jaw via first order neurons of CN V.

**Figure 4.**
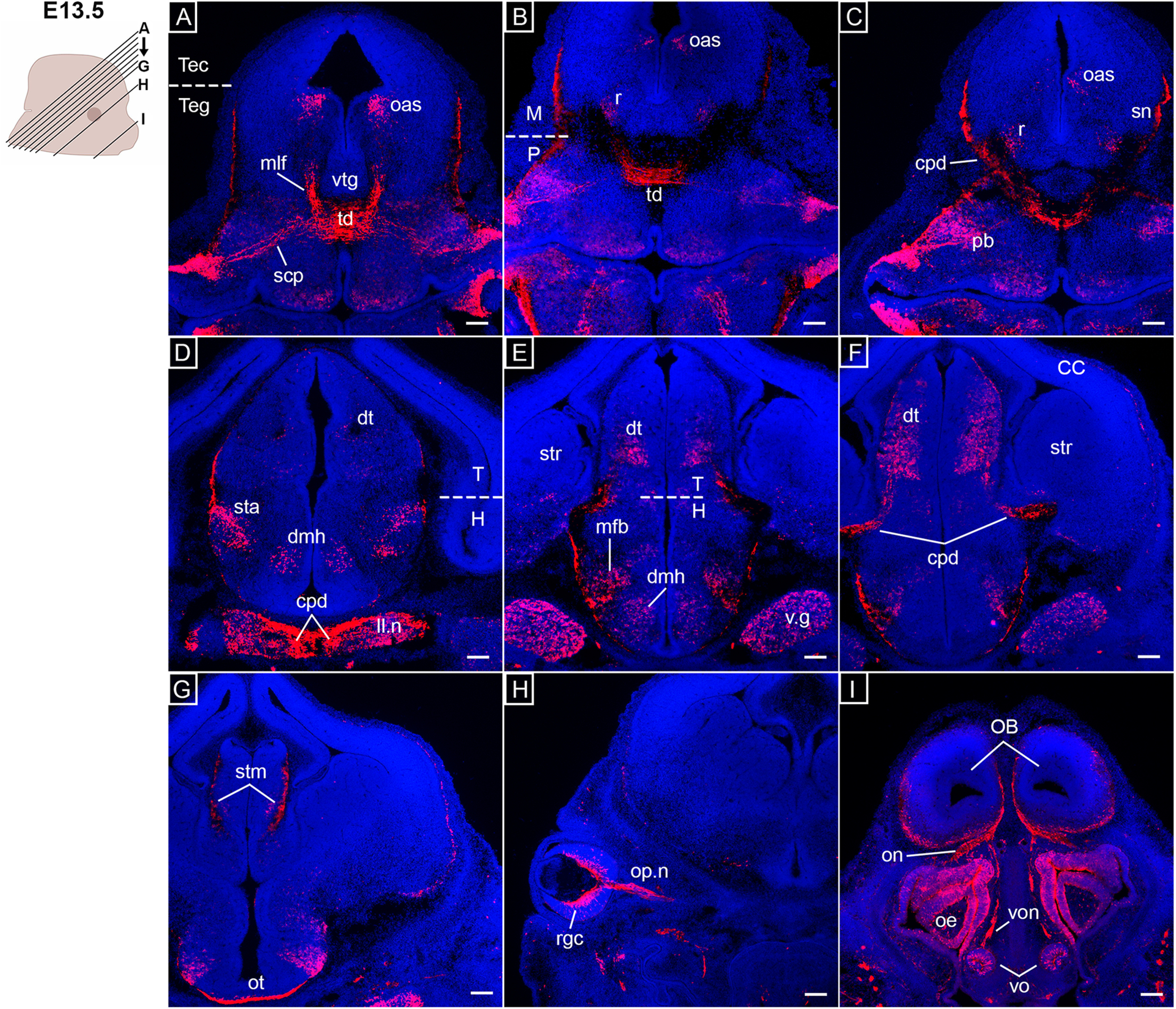
*Opn3*-eGFP immunodetection at E13.5 (red) in coronal serial sections caudo-rostral direction from midbrain to forebrain areas counterstained with DAPI (blue). On the left, a schematic of an E13.5 embryonic head including planes of sections shown in ***A–I***. ***A***, mlf: medial longitudinal fasciculus, oas: oculomotor associated subnuclei, scp: superior cerebellar peduncle, td: tegmental decussations, Tec: tectum of midbrain, Teg: midbrain and pontine tegmentum, vtg: ventral tegmentum. ***B***, M: midbrain, oas: oculomotor associated subnuclei, P: pons, r: red nucleus, td: tegmental decussations. ***C***, cpd: cerebral peduncle, oas: oculomotor associated subnuclei, pb: parabrachial nuclei, r: red nucleus, sn: substantia nigra. ***D***, cpd: cerebral peduncle, dmh: dorsomedial hypothalamic nucleus, dt: dorsal thalamus, H: hypothalamus, ll.n: nucleus of lateral lemniscus, sta: subthalamic area, T: thalamus, v.g: trigeminal ganglion. ***E***, dt: dorsal thalamus, H: hypothalamus, mfb: medial forebrain bundles, str: striatum, T: thalamus, dmh: dorsomedial hypothalamic nucleus**. *F***, CC: cerebral cortex, cpd: cerebral peduncle, dt: dorsal thalamus, str: striatum. ***G***, stm: stria medullaris, ot: optic tract. ***H***, op.n: optic nerve, rgc: retinal ganglion cells. ***I***, OB: olfactory bulb, oe: olfactory epithelia, on: olfactory nerve, vo: vomeronasal organ, von: vomeronasal nerve. Scale bars: 100 μm.

*Opn3-*eGFP expression was also observed in the mesencephalic trigeminal nuclei (Fig. 3*G*), which are involved in both proprioception and motor functions of the jaw. Moreover, *Opn3-*eGFP was detected in the facial nerve (CN VII), including the genu of the facial nerve, which carries both sensory and autonomic fibers (Fig. 3*G*; Extended Data Fig. 1-2*D–F*). By this stage, *Opn3-*eGFP^+^ vagal and glossopharyngeal ganglionic projections have reached the posterolateral region of the medulla to form the solitary tract (Fig. 3*C*), which conveys afferent information from stretch receptors and chemoreceptors in the walls of the cardiovascular and respiratory systems, as well as the gastrointestinal tract. *Opn3-*eGFP was also detected in the parabrachial nuclei (Figs. 3*F*, 4*C*), which relay information from the solitary tract to the thalamus.

At E13.5, *Opn3-*eGFP was detected in both the hypoglossal nucleus and nucleus prepositus (also called nucleus prepositus hypoglossi; Fig. 3*C*,*F*), the latter of which plays an integral role in both vestibular and visual systems by integrating velocity and positional information for horizontal eye movements. *Opn3-*eGFP^+^ vestibular fibers of the vestibulocochlear nerve (CN VIII) reach the medulla where they synapse with medullary vestibular nuclei, also detected as *Opn3-*eGFP^+^ (Fig. 3*C*,*E*,*F*). Furthermore, *Opn3-*eGFP was detected in the medial longitudinal fasciculus (Fig. 3*C*), which form the ascending tract from the superior and medial vestibular nuclei. The medial longitudinal fasciculus terminates in the oculomotor-associated subnuclei that are located along the dorso-ventral and caudo-rostral regions of the tectum of the midbrain (Fig. 4*A–C*), as well as the abducens nucleus in the caudal portion of the pons (Fig. 3*E*). The cochlear nuclei, located in the junction of the pons and medulla where the auditory part of CN VIII synapses, were also identified as *Opn3-*eGFP^+^ (Fig. 3*D–F*). In turn, the medullary cochlear nuclei send information to the superior olivary complex (Fig. 3*D*,*E*), the latter of which are a collection of nuclei important for ascending and descending auditory pathways.

In the cerebellum, *Opn3-*eGFP expression was detected in the external granular layer and deep cerebellar nuclei (Fig. 3*D*,*G*), as well as the superior cerebellar peduncle (Figs. 3*F*, 4*A*). This latter structure mainly consists of cerebellar efferent fibers that via the cerebello-rubral tract project to, among others, the contralateral red nucleus, which also was observed to be *Opn3-*eGFP^+^ (Fig. 4*B*,*C*). Similarly, the tegmental decussations of ascending and descending fibers were *Opn3-*eGFP^+^ (Fig. 4*A*,*B*).

At the level of the forebrain, *Opn3-*eGFP was detected in the subthalamic area and the dorsal thalamus (Fig. 4*D–F*), as were the projections of the cerebral peduncle to the dorsal thalamus and the medial forebrain bundles in the lateral hypothalamus (Fig. 4*D*,*E*). In addition, the dorso-medial and ventro-medial hypothalamic nuclei (Fig. 4*D*,*E*), which are related to the autonomic nervous system, and the stria medullaris (Fig. 4*G*), which is part of the epithalamus, were shown to express *Opn3-*eGFP. At this stage, the developing cerebral cortex did not express *Opn3-*eGFP (Fig. 4*F*). In both visual and olfactory systems, *Opn3-*eGFP was observed in RGCs, the optic nerve and tract (Fig. 4*G*,*H*), as well as the olfactory epithelium, nerve and bulb, and the vomeronasal organ and nerve (Fig. 4*I*).

### Expanded *Opn3*-eGFP expression in sensory organs, tracts, and nuclei important for motor-sensory functions at E15.5

**Figure 5.**
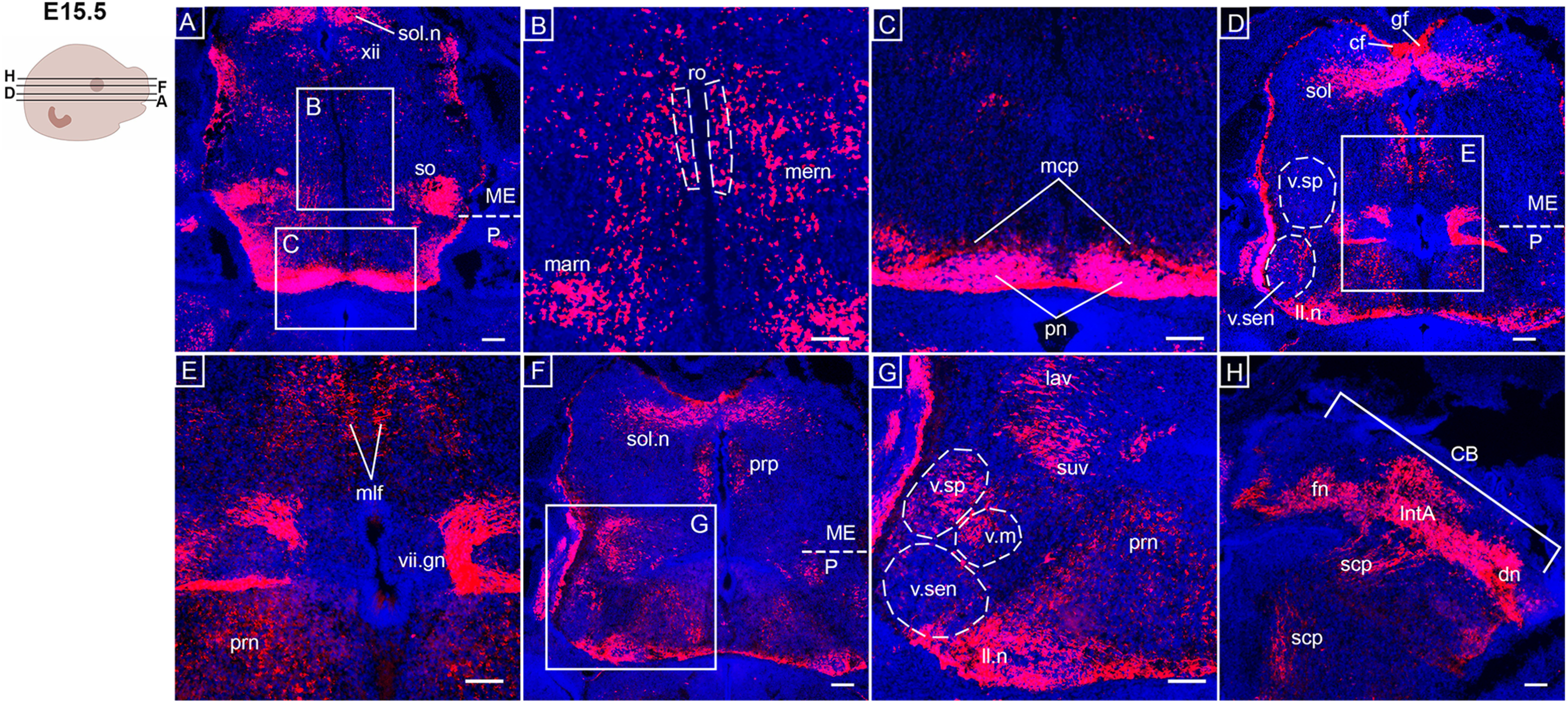
*Opn3*-eGFP immunodetection (red) at E15.5 in horizontal serial sections in caudo-rostral direction from the medulla to pons areas counterstained with DAPI (blue). On the left, a schematic of an E15.5 embryonic head including planes of sections shown in ***A–H***. ***A***, ME, medulla, P: pons, so: superior olivary complex, sol.n: solitary nucleus, xii: hypoglossal nucleus. ***B***, marn: magnocellular reticular nucleus, mern: medullary reticular nuclei, ro: nucleus raphe obscurus. ***C***, mcp: medial cerebellar peduncle, pn: pontine nuclei. ***D***, cf: cuneate fasciculus, gf: gracile fasciculus, ll.n: nucleus of lateral lemniscus, ME: medulla, P: pons, sol: solitary tract, v.sen: sensory trigeminal nucleus, v.sp: spinal trigeminal nucleus. ***E***, mlf: medial longitudinal fasciculus, prn: pontine reticular nuclei, vii.gn: genu of facial nerve. ***F***, ME: medulla, P: pons, prp: nucleus prepositus, sol.n: solitary nucleus. ***G***, lav: lateral vestibular nucleus, ll.n: nucleus of lateral lemniscus, prn: pontine reticular nuclei, suv: superior vestibular nucleus, v.m: motor trigeminal nucleus, v.sen: sensory trigeminal nucleus, v.sp: spinal trigeminal nucleus. ***H***, CB: cerebellum, dn: dentate nucleus, fn: fastigial nucleus, IntA: interposed nucleus, scp: superior cerebellar peduncle. Scale bars: 100 μm.

**Figure 6.**
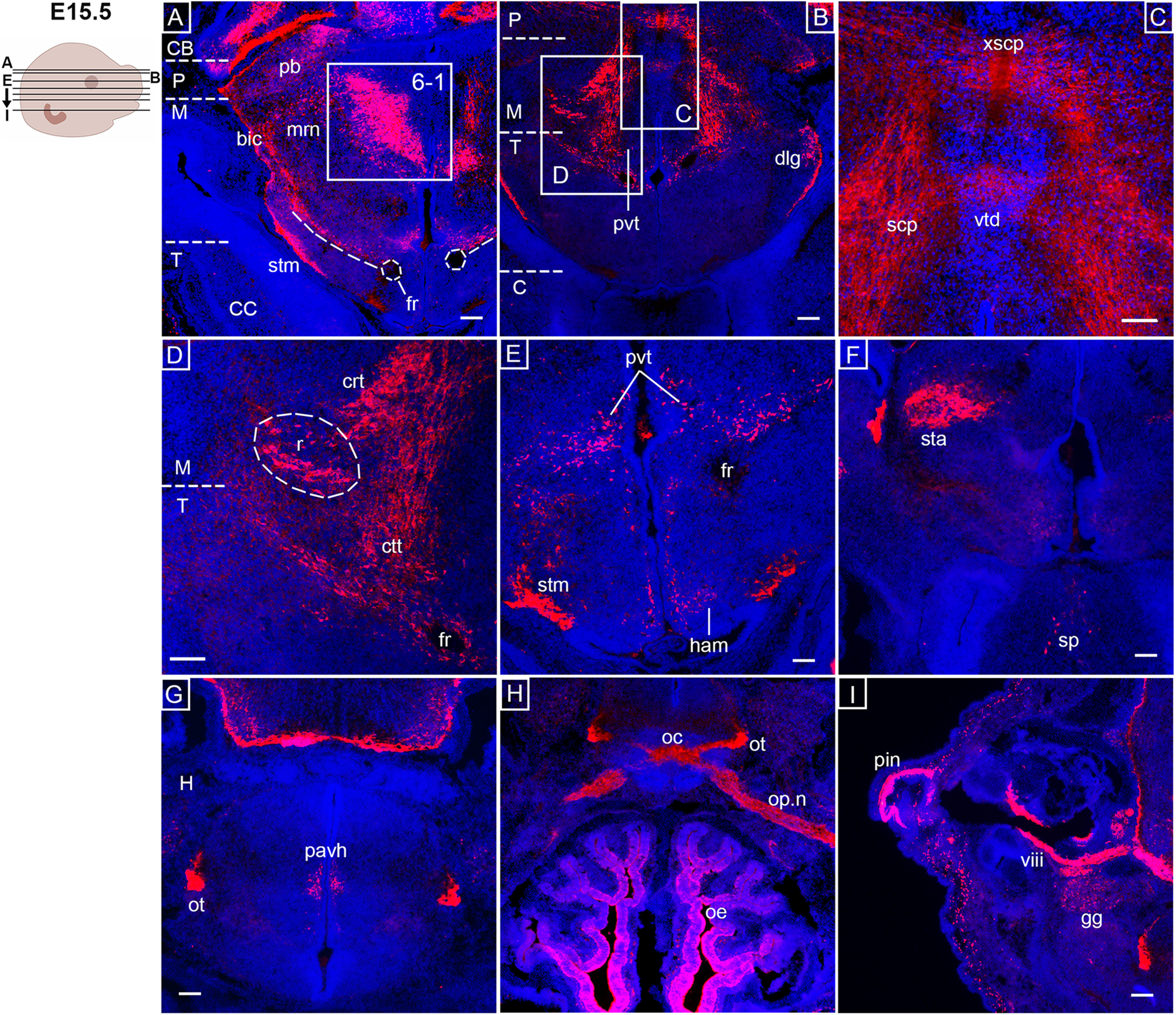
*Opn3*-eGFP immunodetection (red) at E15.5 in horizontal serial sections in caudo-rostral direction from the midbrain to forebrain areas counterstained with DAPI (blue). Left, Schematic of an E15.5 embryonic head including planes of sections shown in ***A–I***. ***A***, bic: brachium of inferior colliculus, CB: cerebellum, CC: cerebral cortex, fr: fasciculus retroflexus, M: midbrain, mrn: midbrain reticular nuclei, P: pons, pb: parabrachial nuclei, stm: stria medullaris, T: thalamus. The inserted box indicates area shown in Extended Data Figure 6-1. ***B***, C: cerebrum, dlg: dorsal lateral geniculate nucleus, M: midbrain, P: pons, pvt: pvt: periventricular thalamic nucleus, T: thalamus. ***C***, scp: superior cerebellar peduncle, vtd: ventral tegmental decussation, xscp: decussation of superior cerebellar peduncle. ***D***, crt: cerebellorubral tract, ctt: cerebellothalamic tract, fr: fasciculus retroflexus, M: midbrain, r: red nucleus, T: thalamus. ***E***, fr: fasciculus retroflexus, ham: medial habenula, pvt: periventricular thalamic nucleus, stm: stria medullaris. ***F***, sp: septal nuclei, sta: subthalamic area. ***G***, H: hypothalamus, ot: optic tract, pavh: paraventricular hypothalamus nucleus. ***H***, oc: optic chiasm, oe: olfactory epithelium, op.n: optic nerve, ot: optic tract. ***I***, gg: geniculate ganglion, pin: pinna of the ear, viii: vestibulocochlear nerve. Scale bars: 100 μm.

At E15.5, additional and expanded *Opn3-*eGFP^+^ regions were observed (Figs. 5, 6). In the medulla, scattered cells expressing *Opn3-*eGFP were located in the medullary reticular nuclei (Fig. 5*A*,*B*), which are related to sleep behavior, and the magnocellular reticular nucleus (Fig. 5*A*,*B*), which project to the branchiomotor nuclei. Moreover, *Opn3-*eGFP expression was observed in the raphe obscurus, which are localized in the midline of the brainstem and involved with autonomic and motor activities (Fig. 5*A*,*B*). Close to the solitary tract, *Opn3-*eGFP was detected in the medullary ascending cuneate and gracile fasciculus (Fig. 5*D*). These fasciculi are part of the dorsal column-medial lemniscus (DCML) pathway, which transfer information about proprioception, exteroception and vibratory sensations from the trunk and extremities. This is consistent with findings at E10.5, where the posterior dorsal root ganglia, which are first order neurons in the DCML pathway, were confirmed to be *Opn3-*eGFP^+^ (Fig. 1*D*,*E*,*J*). Furthermore, *Opn3-*eGFP expression was observed in the medial longitudinal fasciculus located in the lateral wall of the third ventricle (Fig. 5*D*,*E*), as well as the genu of the facial nerve (Fig. 5*D*,*E*) and the nucleus prepositus (Fig. 5*F*). The lateral vestibular nucleus in the upper medulla and the superior vestibular nucleus in the pons, which are both related to the vestibulo-ocular reflex when changing head and eye positions, were shown to express *Opn3-*eGFP (Fig. 5*F*,*G*).

10.1523/ENEURO.0141-21.2021.f6-1 Extended Data Figure 6-1 Confirmation of DAPI labelled nuclei in the region of the oculomotor associated subnuclei. ***A–C***, *Opn3*-eGFP immunodetection (red) at E15.5 in horizontal (***A–C***) sections counterstained with DAPI (blue). ***A***, bic: brachium of inferior colliculus, CB: cerebellum, CC: cerebral cortex, fr: fasciculus retroflexus, M: midbrain, mrn: midbrain reticular nuclei, P: pons, pb: parabrachial nuclei, stm: stria medullaris, T: thalamus. ***B***, oas: oculomotor associated subnuclei. ***C***, 3dv: third ventricle. Scale bars: 100 μm. Download Figure 6-1, TIF file.

In the pons, marked *Opn3-*eGFP expression was detected in the pontine nuclei (also called pontine gray; Fig. 5*A*,*C*) and the medial cerebellar peduncle (Fig. 5*A*,*C*), the latter of which conveys motor information regarding intended movements from the pontine nuclei to the contralateral cerebellum. At E15.5, a clear segregation of the *Opn3-*eGFP^+^ spinal, motor and sensory trigeminal nuclei were observed (Fig. 5*D*,*F*,*G*), as well as noted *Opn3-*eGFP expression in auditory structures such as the nucleus of the lateral lemniscus (Fig. 5*D*,*G*) and the brachium of the inferior colliculus (Fig. 6*A*). The pontine reticular nuclei (Fig. 5*E–G*) and the parabrachial nuclei (Fig. 6*A*), which relay solitary tract information to the forebrain, were detected as *Opn3-*eGFP^+^. In the cerebellum, *Opn3-*eGFP expression was observed in the dentate, fastigial, and interposed nuclei (Fig. 5*H*).

In the midbrain, *Opn3-*eGFP expression was observed in the reticular nuclei (Fig. 6*A*), the oculomotor-associated subnuclei (Fig. 6*A*; Extended Data Fig. 6-1) and the subthalamic area (Fig. 6*F*). *Opn3-*eGFP was also detected in the stria medullaris, containing afferent fibers from, among others, the *Opn3-*eGFP^+^ septal nuclei, projecting to the habenular nuclei in the medial habenula (Fig. 6*A*,*E*,*F*). At this stage, *Opn3-*eGFP expression was identified in the decussation of the superior cerebellar peduncle and the ventral tegmental decussation (Fig. 6*B*,*C*). Consistently, the superior cerebellar peduncle consists of fibers carrying both cerebello-rubral and cerebello-thalamic tracts, here detected as *Opn3-*eGFP^+^ (Fig. 6*B–D*), in which axons project from deep cerebellar nuclei (Fig. 3*G*) to the *Opn3-*eGFP^+^ contralateral red nucleus (Fig. 6*D*) or the ventral lateral nucleus of the thalamus, respectively. Some thalamic nuclei, known to act as relay stations between the midbrain and cortical areas, were also *Opn3-*eGFP^+^; these include the periventricular thalamus nucleus (Fig. 6*B*,*E*) that are associated with the limbic system, as well as the dorsal lateral geniculate nucleus (Fig. 6*F*), which is related to the visual pathway. Other central projections of the visual system were clearly labeled as *Opn3-*eGFP^+^, including the optic nerve, chiasm and tract (Fig. 6*G*,*H*), where visual information is transferred to the dorsal lateral geniculate nucleus. *Opn3-*eGFP expression was also observed in the olfactory sensory epithelium (Fig. 6*H*) and in parts of the auditory system, namely the pinna of the ear, vestibular nerve and geniculate ganglion (Fig. 6*I*). In addition, scattered *Opn3-*eGFP expression was also noted in the septal nuclei (Fig. 6*F*) and the paraventricular hypothalamic nucleus (Fig. 6*G*).

### Several *Opn3*-eGFP-positive forebrain nuclei are observed at E17.5

**Figure 7.**
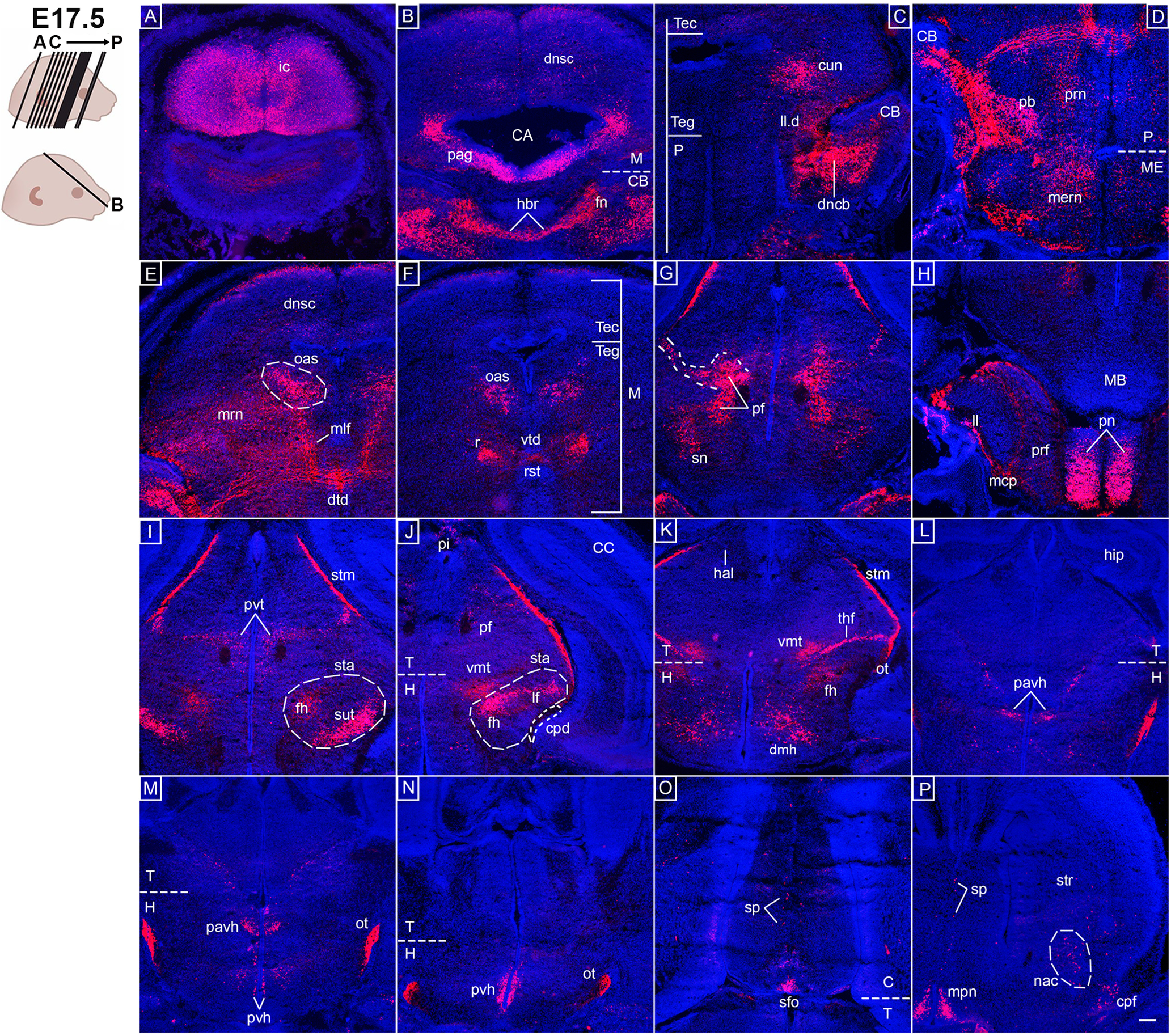
*Opn3*-eGFP immunodetection (red) at E17.5 in horizontal (***B***) and coronal (***A***, ***C–P***) serial sections from the hindbrain to forebrain areas counterstained with DAPI (blue). Left, Schematic of E17.5 embryonic heads including planes of sections shown in ***A–P***. ***A***, ic: inferior colliculus. ***B***, CA: cerebral aqueduct, CB: cerebellum, dnsc: deep nuclei of superior colliculus, fn: fastigial nucleus, hbr: hooked bundle of Russel, M: midbrain, pag: periaqueductal gray. ***C***, CB: cerebellum, cun: cuneiform nucleus, dncb: deep nuclei of cerebellum, ll.d dorsal component of lateral lemniscus, P: pons, Tec: tectum of midbrain, Teg: tegmentum of midbrain. ***D***, CB: cerebellum, ME: medulla, mern: medullary reticular nuclei, P: pons, pb: parabrachial nuclei, prn: pontine reticular nuclei. ***E***, dnsc: deep nuclei of superior colliculus dtd: dorsal tegmental decussation, mlf: medial longitudinal fasciculus, mrn: midbrain reticular nuclei, oas: oculomotor associated subnuclei. ***F***, M: midbrain, oas: oculomotor associated subnuclei, r: red nucleus, rst: rubrospinal tract, Tec: tectum of midbrain, Teg: tegmentum of midbrain, vtd: ventral tegmental decussation. ***G***, sn: substantia nigra, pf: parafascicular nucleus. ***H***, ll: lateral lemniscus, MB: mamillary body, mcp: medial cerebellar peduncle, pn: pontine nuclei (gray), prf: pontine reticular formation. ***I***, fh: field ***H***, pvt: paraventricular thalamic nucleus, sta: subthalamic area, stm: stria medullaris, sut: subthalamic nucleus. ***J***, CC: cerebral cortex, cpd: cerebral peduncle, fh: field ***H***, H: hypothalamus, lf: lenticular fasciculus, pf: parafascicular nucleus, π: pineal gland, sta: subthalamic area, T: thalamus, vmt: ventromedial thalamic nucleus. ***K***, fh: field ***H***, H: hypothalamus, hal lateral habenula, ot: optic tract, stm: stria medullaris, T: thalamus, thf: thalamic fasciculus, dmh: dorsomedial hypothalamic nucleus, vmt: ventromedial thalamic nucleus. ***L***, hip: hippocampus, H: hypothalamus, pavh: paraventricular hypothalamic nucleus, T: thalamus. ***M***, H: hypothalamus, ot: optic tract, pavh: paraventricular hypothalamic nucleus, pvh periventricular hypothalamic nucleus, T: thalamus. ***N***, H: hypothalamus, ot: optic tract, pvh periventricular hypothalamic nucleus, T: thalamus. ***O***, C: cerebrum, sfo: subfornical organ, sp: septum of brain, T: thalamus. ***P***, nac: nucleus accumbens, cpf: pyriform (olfactory) cortex, mpn: medial preoptic nucleus, sp: septum of brain, str: striatum. Scale bar: 100 μm. Extended Data Figure 7-1 is supporting this figure.

Additional *Opn3-*eGFP^+^ structures were more clearly detected at E17.5 compared with earlier stages. Specifically, *Opn3-*eGFP expression was observed in the inferior colliculus (Fig. 7*A*), which receives auditory input from the superior olivary complex (Fig. 5*A*) via the lateral lemniscus (Fig. 7*C*,*H*), which was also observed to be *Opn3-*eGFP^+^. Moreover, the periaqueductal gray (Fig. 7*B*), which plays a critical role in the autonomic system, motivated behavior and pain modulation, was *Opn3-*eGFP^+^. In the cerebellum, the fastigial nucleus was *Opn3-*eGFP^+^, as was the uncinate fasciculus of the cerebellum (also known as the hooked bundle of Russell; Fig. 7*B*), which consists of fibers that descend to the superior cerebellar peduncle. In the midbrain, the cuneiform nucleus and midbrain reticular nuclei (Fig. 7*C*,*E*), both included in the midbrain reticular formation, were identified as *Opn3-*eGFP^+^. Other *Opn3-*eGFP^+^ midbrain-located nuclei comprised the deep nuclei of the superior colliculus (Fig. 7*B*,*E*) and the parafascicular nucleus (Fig. 7*G*,*J*), the latter of which is considered to be an essential thalamic structure in the feedback systems of basal ganglia-thalamo-cortical circuits involved in cognitive processes. At this stage, the rubro-spinal tract, which originates from the red nucleus and crosses the midline via the ventral tegmental decussation, were all detected by *Opn3-*eGFP (Fig. 7*F*), as was the dorsal tegmental decussation (Fig. 7*E*). Furthermore, *Opn3-*eGFP expression was observed in the substantia nigra (Fig. 7*G*), a basal ganglia structure, and in the three white matter areas of the subthalamus, including the lenticular fasciculus, thalamic fasciculus and field H (Fig. 7*I–K*), in addition to the subthalamic nucleus (Fig. 7*I*).

10.1523/ENEURO.0141-21.2021.f7-1 Extended Data Figure 7-1 *Opn3*-eGFP expression was not observed in GFAP^+^ astrocytes. On the left, a schematic of an E17.5 head including planes of sections shown in ***A–D***. ***A–D***, *Opn3*-eGFP (red) and GFAP (green) immunodetection at E17.5 in coronal sections counterstained with DAPI (blue). *Opn3*-eGFP and GFAP was not co-expressed in GFAP^+^ astrocytes in the hippocampal, thalamic or hypothalamic regions. ***C***, ***D***, Minimal co-localization, but not co-expression, of *Opn3*-eGFP and GFAP was observed in parts of the optic tract. ***A***, gl: glia limitans, Hip: hippocampus, stm: stria medullaris. ***B***, dg: dentate gyrus, fim: fimbria, stm: stria medullaris. ***C***, 3dv: third ventricle, dg: dentate gyrus, fim: fimbria, ot: optic tract, pvt: paraventricular thalamic nucleus, stm: stria medullaris, vmt: ventromedial thalamic nucleus. ***D***, 3dv: third ventricle, mpn: medial preoptic nucleus, ot: optic tract, pavh: paraventricular hypothalamic nucleus, pvh periventricular hypothalamic nucleus. Scale bars: 100 μm. Download Figure 7-1, TIF file.

At E17.5, *Opn3-*eGFP was detected in the pineal gland (Fig. 7*J*), an important structure well known to be involved in photoreception by producing melatonin during the dark phase of the sleep-wake cycle, as well as the ventro-medial thalamic nucleus (Fig. 7*J*,*K*), which projects pain-temperature information to the insular cortex. In the forebrain, *Opn3-*eGFP expression was observed in several hormone-releasing nuclei; namely the paraventricular and periventricular hypothalamic nuclei (Fig. 7*L–N*), and the medial preoptic nucleus (Fig. 7*P*), which all remained *Opn3-*eGFP^+^ at postnatal/adult stages (Extended Data Fig. 7-1*B*,*F*,*H*). Other *Opn3-*eGFP*^+^* regions included the pyriform (olfactory) cortex (Fig. 7*P*); the subfornical organ (Fig. 7*O*), which is one of the circumventricular organs of the brain; and the nucleus accumbens (Fig. 7*P*), which is part of the reward and reinforcement systems. Moreover, scattered *Opn3-*eGFP expression was observed throughout the septum of the brain (Fig. 7*O*,*P*).

Studies of adult Opn3::mCherry reporter mice have reported that Opn3 is expressed also in non-neuronal astrocytes (Olinski et al., 2020b). In mouse, the differentiation of astrocytes begins ∼E17–E18 (Reemst et al., 2016; Matsue et al., 2018). To examine whether Opn3 is expressed in astrocytes at embryonic stages, double-immunohistochemistry of *Opn3*-eGFP and GFAP, a known astrocyte marker (Reemst et al., 2016), was performed at E17.5. At this stage, GFAP^+^ astrocytes were identified in the hippocampus, in particular the dentate gyrus and the fimbria, and around the third ventricle at the level of the paraventricular hypothalamic nucleus, but GFAP was not co-expressed with *Opn3*-eGFP (Extended Data Fig. 7-1*A–D*). Moreover, GFAP^+^ astrocytes were observed along the optic tract, in the glia limitans, the granular layer of olfactory bulb, septohippocampal nucleus, habenula, some regions of the stria medullaris and pyramidal tract, but again not co-expressed with *Opn3*-eGFP (Extended Data Fig. 7-1*A*,*C*,*D*; data not shown). Thus, these results indicate that *Opn3*-eGFP is not expressed in GFAP^+^ astrocytes in the embryonic brain.

### Expanded *Opn3*-eGFP expression in the brain at postnatal and adult stages

**Figure 8.**
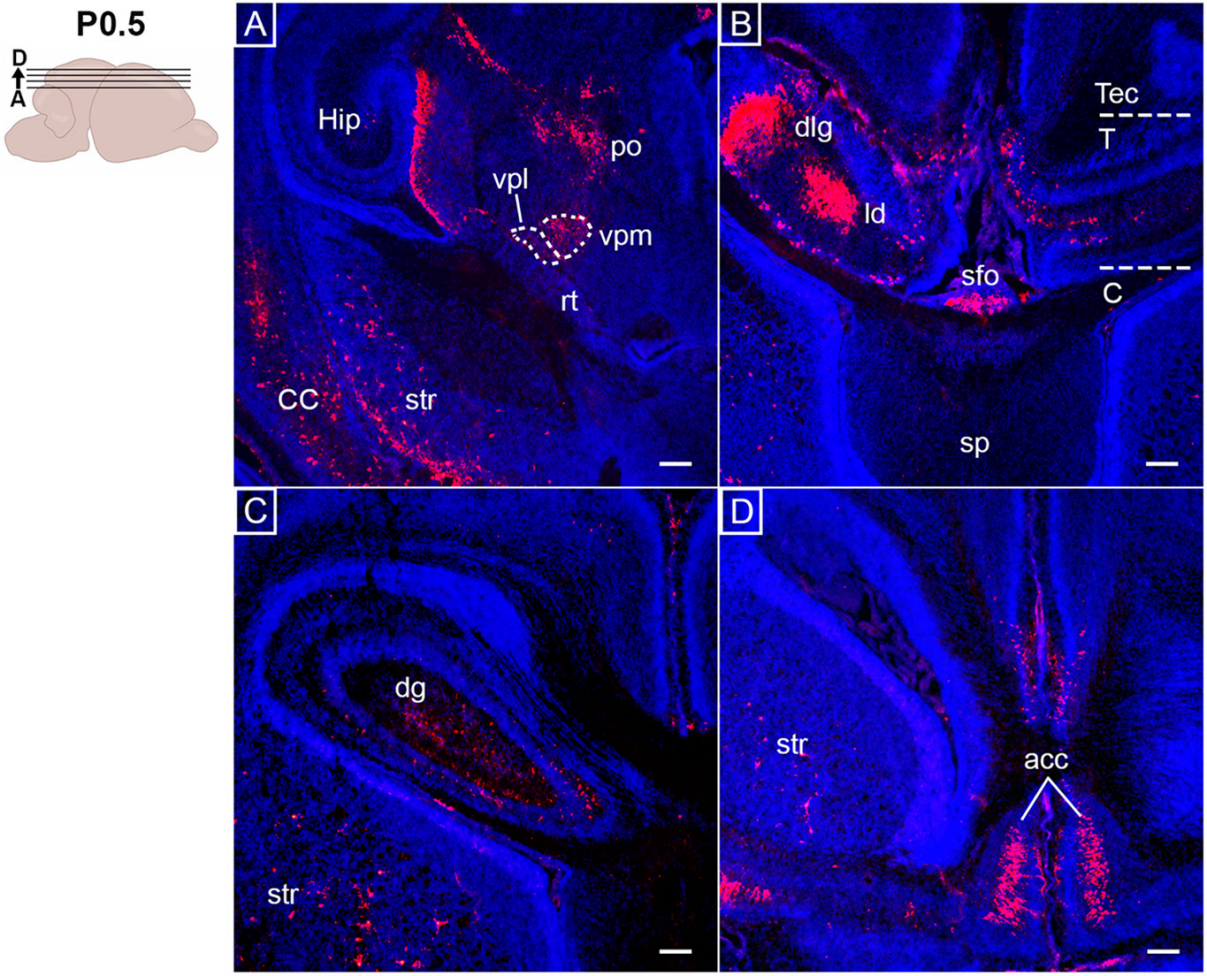
*Opn3*-eGFP immunodetection (red) at P0.5 in horizontal sections of forebrain areas counterstained with DAPI (blue). On the left, a schematic of a P0.5 brain including planes of sections shown in ***A–D***. ***A***, CC: cerebral cortex, Hip: hippocampus, p: posterior thalamus, rt: thalamic reticular nucleus, str: striatum, vpl: ventral posterolateral nucleus, vpm: ventral posteromedial nucleus. ***B***, C: cerebrum, dlg: dorsal lateral geniculate nucleus, ld: lateral dorsal nucleus, sfo: subfornical organ, sp: septal nuclei, T: thalamus, Tec: tectum of midbrain. ***C***, dg: dentate gyrus, str: striatum. ***D***, acc: anterior cingulate cortex, str: striatum. Scale bars: 100 μm. Extended Data Figure 8-1 is supporting this figure.

**Figure 9.**
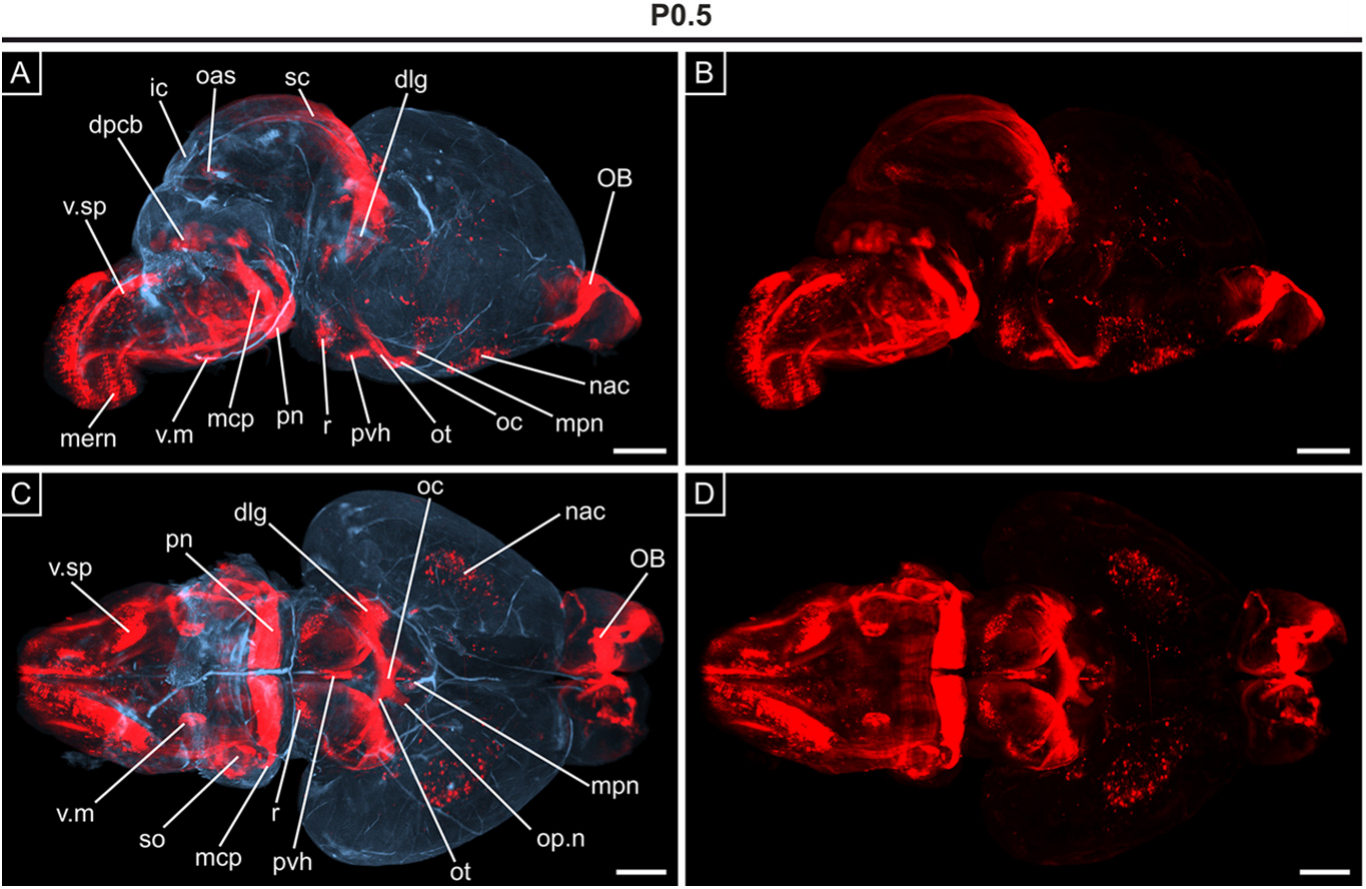
3D OPT imaging of *Opn3*-eGFP immunodetection in the brain at P0.5. ***A***, Sagittal view showing the presence of *Opn3*-eGFP (red) against a background of auto-fluorescing anatomic structures (blue). ***B***, Repeat of (***A***) showing unmasked *Opn3*-eGFP (red) staining only. ***C***, ***D***, Ventral views of the brain shown in ***A***, ***B***, respectively. dlg: dorsal lateral geniculate nucleus, dpcb: deep nuclei of cerebellum, ic: inferior colliculus, mcp: medial cerebellar peduncle, mern: medullary reticular nuclei, mpn: medial preoptic nucleus, nac: nucleus accumbens, oas: oculomotor associated subnuclei, OB: olfactory bulb, oc: optic chiasm, op.n: optic nerve, ot: optic tract, pn: pontine nuclei (also pontine nuclei gray), pvh: periventricular hypothalamic nucleus, r: red nucleus, sc: superior colliculus, so: superior olivary complex, v.m: motor trigeminal nucleus, v.sp: spinal trigeminal nucleus. Scale bars: 1 mm.

Just after birth, at P0.5, an expansion of *Opn3-*eGFP expression was observed in the forebrain, including the cerebral cortex, with defined expression in the anterior cingulate cortex, involved in higher-level social functions (Fig. 8*A*,*D*), which was even more pronounced at P10 (Extended Data Fig. 8-1*D*). At P0.5, *Opn3-*eGFP expression was also noted in the striatum and in parts of the hippocampus, including the dentate gyrus (Fig. 8*A*,*C*). Moreover, *Opn3-*eGFP^+^-thalamic regions were identified, including the posterior thalamus, lateral dorsal nucleus, as well as ventral posteromedial and lateral nuclei (Fig. 8*A*,*B*). Notably, the ventral posterolateral nucleus of the thalamus (Fig. 8*A*) receives information from, among others, the DCML pathway, which incorporated several *Opn3-*eGFP^+^ structures detected at E15.5 (Fig. 5*D*). Also, *Opn3-*eGFP was detected in the dorsal lateral geniculate nucleus (Fig. 8*B*), which is a relay center for visual information from the optic nerve. At this stage, OPT imaging, to appreciate the 3D perspective, clearly confirmed the expression of *Opn3-*eGFP in the medulla and pons, optic chiasm and tracts, dorsal lateral geniculate nuclei and areas of the primary visual cortex, as well as the olfactory bulb and several hypothalamic nuclei (Fig. 9; Movie 2).

10.1523/ENEURO.0141-21.2021.f8-1 Extended Data Figure 8-1 *Opn3*-eGFP immunodetection (red) at P10 in horizontal (***A–D***) and at adult stage in coronal (***E–H***) serial sections of forebrain areas counterstained with DAPI (blue). On the left, schematics of P10 and adult brains including planes of sections shown in A-H. ***A***, MB: mammillary bodies, mh: medial hypothalamic nuclei. ***B***, fx: posterior fibers of the fornix, pavh: paraventricular hypothalamic nucleus, ph: posterior hypothalamus, sn: substantia nigra. ***C***, dg: dentate gyrus, dlg: dorsal lateral geniculate nucleus, EC: entorhinal cortex, mg: medial geniculate nucleus, om: outer membrane, sub: subiculum. ***D***, acc: anterior cingulate cortex, CC: cerebral cortex, fx: fibers of fornix, hc: hippocampal commissure, ld: lateral dorsal thalamic nucleus, sfo: subfornical organ, str: striatum, sp: septal nuclei. ***E-H***, *Opn3*-eGFP immunodetection using an anti-rabbit 488 antibody, here pseudo-colored (from green to red). ***E***, dmh: dorsomedial hypothalamic nucleus, fx: fibers of the fornix, pfn: perifornical lateral hypothalamic nuclei, tn: tanycytes. ***F***, pavh: paraventricular hypothalamic nucleus, pvh: periventricular hypothalamic nucleus. ***G***, son.rx: retrochiasmatic supraoptic nucleus. ***H***, bnst: bed nucleus of stria terminalis, mepn: median preoptic nucleus, mpn: medial preoptic nucleus, son.p: supraoptic nucleus proper. Scale bars: 100 μm. Download Figure 8-1, TIF file.

Movie 2. Video of 3D imaging of a P0.5 brain (along the *x*-axis) showing *Opn3*-eGFP immunodetection (red) against a background of auto-fluorescing anatomical structures (blue). 10.1523/ENEURO.0141-21.2021.video.2

Additional *Opn3-*eGFP^+^ regions were identified at P10, namely the mammillary bodies and medial hypothalamic nuclei (Extended Data Fig. 8-1*A*), which are important for memory. Other parts of the limbic system that express *Opn3-*eGFP included the anterior and posterior fibers of the fornix (Extended Data Fig. 8-1*B*,*D*,*E*), which connect the hippocampus with the forebrain. Furthermore, part of the cortico-striatal circuitry, between the anterior cingulate cortex and the striatum, which includes the reward system, were *Opn3-*eGFP^+^ (Extended Data Fig. 8-1*D*). The posterior hypothalamus was also noted to express *Opn3-*eGFP (Extended Data Fig. 8-1*B*). The entorhinal cortex, important for spatial memory in relation to the hippocampus, was *Opn3-*eGFP^+^, as were the dentate gyrus and subiculum of the hippocampal formation (Extended Data Fig. 8-1*C*,*D*). In the thalamus, *Opn3-*eGFP expression was observed in the medial geniculate nucleus and the dorsal lateral geniculate nucleus (Extended Data Fig. 8-1*C*), which are related to auditory and visual pathways, respectively. The outer membrane, surrounding the brain also expressed *Opn3-*eGFP (Extended Data Fig. 8-1*C*).

During adulthood, *Opn3-*eGFP expression was identified in the perifornical lateral hypothalamic nuclei, which are related to respiratory control, and in α-tanycytes that line the lateral walls of the third ventricle (Extended Data Fig. 8-1*E*). Furthermore, *Opn3-*eGFP was identified in the hormone-secreting supraoptic nucleus of the adult hypothalamus, including both the supraoptic nucleus proper and retro-chiasmatic supraoptic nucleus, and in the bed nucleus of the stria terminalis, which is a structure related to stress responses (Extended Data Fig. 8-1*G*,*H*). A few examples of areas that expressed *Opn3-*eGFP already at embryonic stages that also were identified at early postnatal stages and in the adult brain, were the substantia nigra, the paraventricular and periventricular hypothalamic nuclei, the dorsal lateral geniculate nucleus, the septal nuclei, the dorsomedial hypothalamic nucleus and the preoptic area (Extended Data Fig. 8-1*B–F*).

## Discussion

Whereas most visual and non-visual opsins appear to be expressed in more or less restricted patterns, *Opn3* has been shown to be expressed in a broad range of multiple adult tissues (Blackshaw and Snyder, 1999; Nissilä et al., 2012; Olinski et al., 2020b). *Opn3* mRNA was first discovered in the cerebral cortex and cerebellum of the adult mouse brain (Blackshaw and Snyder, 1999), results that have been subsequently verified and expanded, most recently in studies using an *Opn3*::mCherry mouse model (Olinski et al., 2020b). Despite more detailed knowledge about the spatial expression of *Opn3* in the adult mouse brain, there is a significant lack of information concerning when and where in the nervous system *Opn3* is expressed during embryogenesis.

To address this issue, an *Opn3* promoter-driven eGFP mouse line was used to investigate *Opn3* expression in the developing nervous system with a focus on the brain/head region. A summary of *Opn3*-eGFP^+^ key structures in relation to temporal onset of expression is outlined in Table 1. Notably, our study identified 25 new *Opn3*-eGFP^+^ structures in the CNS and PNS, including sensory ganglions, the olfactory epithelium, vestibular nuclei and parafascicular nucleus, that have not been previously reported to express Opn3 (Table 1). The analyzed *Opn3*-eGFP knock-in mouse line possesses two intact *Opn3* wild-type alleles that are consistent with an observed normal phenotype, which is in contrast to the recently developed Opn3::mCherry fusion protein mouse line (Olinski et al., 2020b). Being under the influence of the native *Opn3* promoter, both detected eGFP and Opn3::mCherry expression patterns appear to mirror the cellular expression profile of endogenous Opn3 from earlier reports. Moreover, *Opn3*-eGFP cellular expression in the adult brain, as characterized by this current study, mirrors that shown for Opn3::mCherry (Olinski et al., 2020a), which implicates that both *Opn3* reporter mice are accurately detecting Opn3-positive cells. As eGFP is a cytoplasmic protein, the reporter molecule is free to diffuse throughout the cell. As such, not only cell bodies, but also axonal projections are readily visible in the *Opn3*-eGFP reporter mouse line used here, which is greatly informative when analyzing anatomic-functional relationships.

**Table 1.** A Summary outlining the onset of *Opn3*-eGFP expression in various structures of the CNS and PNS, indicating novel *Opn3*-eGFP^+^ structures identified in this study (•)

PNS and sensory organs	CNS		Age
Trigeminal ganglion •	Neural tube	Basal plate	E9.5
Facio-acoustic complexganglion •	Brainvesicles	Basal plate of diencephalon	
Olfactory placode		Dorsal outer cells of mesencephalon	
Dorsal root ganglion	Spinal cord	Motor neurons	E10.5
Inferior vagus ganglion •			
Glossopharyngealganglion •	Brainvesicles	Optic stalk	
Migratory olfactoryneurons			
Retinal ganglion cells	Spinal cord	Dorsal, lateral and ventral mantleand marginal layers	E13.5
Optic nerve			
Olfactory epithelium •	Hindbrain	Spinal trigeminal nucleus •	
Olfactory nerve •		Principal sensory trigeminal nucleus •	
Vomeronasal organ •		Mesencephalic trigeminal nucleus •	
Vomeronasal nerve •		Genu of facial nerve •	
		Solitary tract	
		Parabrachial nuclei •	
		Hypoglossal nucleus •	
		Nucleus prepositus •	
		Vestibular nuclei •	
		Medial longitudinal fasciculus	
		Abducens nucleus •	
		Cochlear nuclei •	
		Superior olivary complex	
		External granular layers of cerebellum	
		Deep cerebellar nuclei	
		Superior cerebellar peduncle	
	Midbrain	Red nucleus	
		Oculomotor associated sub nuclei •	
		Tegmental fibres	
	Forebrain	Subthalamic area	
		Dorsal thalamus	
		Dorsal medial hypothalamic nucleus	
		Stria medullaris	
		Optic tract	
		Olfactory bulb	
Pinna of the ear	Hindbrain	Medullary reticular nuclei	E15.5
		Magnocellular reticular nucleus	
		Raphe obscurus •	
		Cuneate and gracile nuclei •	
		Pontine nuclei	
		Medial cerebellar peduncle •	
		Motor trigeminal nucleus	
		Nucleus of lateral lemniscus	
		Brachium of inferior colliculus	
	Midbrain	Midbrain reticular nuclei	
		Inferior colliculus	
		Brachium of inferior colliculus	
	Forebrain	Periventricular thalamic nucleus	
		Dorsal lateral geniculate nucleus	
		Septal nuclei •	
		Paraventricular hypothalamic nucleus	
	Cerebellum	Uncinate fasciculus of cerebellum	E17.5
	Midbrain	Periaqueductal gray •	
		Cuneiform nucleus	
		Rubrospinal tract	
		Substantia nigra	
	Epithalamus	Pineal gland	
	Thalamus	Parafascicular nucleus •	
		Ventromedial thalamic nucleus	
	Subthalamus	Subthalamic nucleus	
	Hypothalamus	Periventricular hypothalamic nucleus	
		Medial hypothalamic nucleus	
	Cerebrum	Subfornical organ	
		Nucleus accumbens	
		Pyriform cortex	
	Thalamus	Posterior thalamus	P0.5
		Lateral dorsal nucleus	
		Ventral posteromedial nucleus	
		Ventral posterolateral nucleus	
	Subpallium	Dentate gyrus of hippocampus	
	Pallium	Anterior cingulate cortex	
		Frontal cortex	
	Hypothalamus	Mammillary nuclei	P10
		Posterior nucleus	
	Subpallium	Fornix	
		Subiculum	
		Corticostrial circuitry	
	Meninges	Outer membrane	
	Hypothalamus	Perifornical nuclei	Adult
		Tanycytes	
		Supraoptic nucleus	
		Bed nucleus of stria terminalis	

In this study, evidence is provided that *Opn3* is expressed during most of embryonic development, and *Opn3*-eGFP was detected already at E9.5, much earlier than previously reported, in structures within the CNS and PNS. This timeline of expression indicates that *Opn3* may be the earliest of the opsin family of genes to be expressed in mammalian development. At E9.5–E10.5, *Opn3*-eGFP expression was detected in most, if not all, spinal nerves, their associated dorsal root ganglia, as well as several sensory ganglia, all of which are part of the PNS. The sensory ganglia form first order sensory neurons projecting to somatic tissues via ascending sensory tracts to brainstem and forebrain regions, while the spinal cord motor neurons form first order motor neurons connected directly to muscles (Chen et al., 2017). Results presented here show *Opn3*-eGFP expression in motor neurons and spinal nerves at E10.5, before observed *Opn3*-eGFP expression in sensory neurons at E13.5, which are in agreement with the temporal differentiation of first order spinal motor neurons before sensory neurons (Rodier, 1980).

Following embryonic growth, the temporal onset of O*pn3*-eGFP expression correlated with the development of individual neural structures in specific pathways and circuits (Chen et al., 2017), which indicates a potential role for O*pn3* in early neural development and subsequent maturation. Among the different sensory systems, the olfactory-associated pathway is the first to express O*pn3*-eGFP, with expression observed in the olfactory placode at E9.5, followed by postmitotic migratory neurons at E10.5, the nerve and bulb at E13.5 and the olfactory cortex at E17.5. *Opn3* is also expressed in the visual system, where *Opn3*-eGFP was evident in the optic stalk at E10.5, as well as RGCs at E13.5, including their *Opn3*-eGFP^+^ axons in the optic nerve. Consistently, a previous study demonstrated by RT-PCR that *Opn3* is expressed at E10.5 during ocular development in mouse (Tarttelin et al., 2003a). The optic nerves send central projections to the dorsal lateral geniculate nucleus of the thalamus, in this study detected by *Opn3*-eGFP from E15.5, and to the primary visual cortex, shown to express Opn3 at adulthood (Olinski et al., 2020b). From E13.5 to adult stages, *Opn3*-eGFP expression expands throughout the spinal cord, brainstem and forebrain. Ventral, intermediate and dorsal gray matters in the spinal cord were identified as *Opn3*^+^ regions that form motor, autonomic and sensory spinal cord components. Moreover, the onset of *Opn3*-eGFP in the hypothalamic paraventricular nucleus ∼E15.5, was followed by the detection of *Opn3*-eGFP in the periventricular nucleus, dorsomedial hypothalamic nucleus and the medial preoptic nucleus, all of which were obvious at E17.5. Moreover, *Opn3*-eGFP expression was noted in the basal ganglia at E15.5, which connects to multiple thalamic nuclei with clear expression at E17.5, including the parafascicular nucleus, a part of the medial and intralaminar nuclei that is hypothesized to play a critical role in the sensorimotor process of attentional orienting and behavioral flexibility (Brown et al., 2010; Yamanaka et al., 2018). Other *Opn3*-eGFP^+^ subthalamic structures that are related to the basal ganglia, and involved in modulated motor control, include the white and gray matters of the fields of Forel, a structure implicated in epilepsy, movement and behavioral disorders (Neudorfer and Maarouf, 2018), as well as the subthalamic nucleus. All of the above are examples of that *Opn3*-eGFP onset of expression follows the individual developmental maturation of these neural structures.

In brief, thalamic nuclei connect various CNS structures with the cerebral cortex. Interestingly, no *Opn3*-eGFP expression was detected in association with the thalamic nuclei until after birth at P0.5. Consistently, the cerebral cortex was also *Opn3*-negative before birth, with cortical *Opn3*-eGFP expression coinciding with the onset of expression in the thalamic lateral dorsal, ventral posteromedial and ventral posterolateral nuclei at P0.5. In the cerebral cortex, observed *Opn3*-eGFP^+^ areas included the pyriform cortex (∼E17.5), cingulate cortex and archicortex (the hippocampus and associated structures; ∼P0.5), and the entorhinal cortex (∼P10). In subcortical areas, *Opn3*-eGFP was detected in the septal nuclei and nucleus accumbens at ∼E17.5. Other extra-cortical structures that form part of limbic system were also shown to express *Opn3*-eGFP, namely the hypothalamic mammillary bodies (∼P10) and the ventral medial thalamic nucleus (∼E17.5). Consistently, previous reports have shown that *Opn3* is detectable in the cingulate, insula and frontal cortical regions of the adult brain, mainly in layer V pyramidal neurons that are characterized mainly by their cortico-striatal projections (Blackshaw and Snyder, 1999; Olinski et al., 2020b). These latter structures are required for appropriate goal-directed behaviors, including motivation and cognitive processes that mediate appropriate actions to obtain a specific outcome (Gerfen et al., 2013; Haber, 2016). As such, the presence of *Opn3*-eGFP in limbic-associated regions suggests potential (photoreceptive) roles in motivation, emotion, learning and memory.

The absence of *Opn3*-eGFP expression is also of functional significance and importance. In particular, *Opn3*-eGFP was not detected in the cerebral crus that carry cortico-spinal, cortico-bulbar (brainstem), and cortico-pontine tracts. As such, these data suggest that cortical nuclei expressing *Opn3*-eGFP in the frontal cortex are part of the inter-telencephalic cortico-striatal circuitry, with the exclusion of pyramidal neural network. Thus, *Opn3* is most likely restricted to either the premotor and/or supplementary motor areas in the frontal cortex, where all cortico-striatal connections are required before facilitating movement.

By using *Opn3*-eGFP as a cytoplasmic reporter, it was possible to track and trace neuronal cell bodies and their axonal projections for all *Opn3*^+^ cell types. As a result, *Opn3*-eGFP was detected in the dorsal, lateral and ventral marginal layers that form the nerves of the second order DCML sensory tract, including both gracile and cuneate fasciculi, as well as spinothalamic, spinocerebellar, spino-tectal, spino-reticular, and spino-vestibular tracts. These data suggest a possible involvement of *Opn3* in modulating sensory information pertaining to proprioception and fine touch. Also, from E13.5, the presence of *Opn3*-eGFP in higher CNS structures located in the brainstem identified second order ascending (brainstem to forebrain) and descending (brainstem to spinal cord) tracts. These include: (1) the trigeminal lemniscus (carrying sensory information from sensory and spinal trigeminal nuclei to the ventral posteromedial nucleus of the thalamus); (2) the vestibulo-ocular tract (from medullary vestibular nuclei to the oculomotor associated subnuclei via the medial longitudinal fasciculus); and (3) tracts of the auditory system (from the cochlear nucleus to the superior olivary complex, followed by the lateral lemniscus, then the inferior colliculus, and finally the medial geniculate nucleus). Further tracts identified by *Opn3*-eGFP were: (4) the solitary-parabrachial tract (carrying sensory information from CN VII, CN IX, and CN X to the solitary nucleus and parabrachial nuclei); (5) the cerebellar peduncles [including the superior (deep cerebellar nuclei projections), medial (pontine nuclei projections), and inferior (spino-cerebellar tract)]; and (6) the cerebral peduncle that mainly carries sensory input to the forebrain. Together these observations emphasize that studies of *Opn3*-eGFP expression can be used to follow projections for various neural systems and that *Opn3* appears to play a role in the development of multiple sensory pathways.

Given that Opn3 has been shown to functionally form a short-wavelength-sensitive photopigment, with a spectral peak of absorbance in the blue range (Sugihara et al., 2016), the widespread expression pattern of *Opn3* during embryogenesis and well into adulthood suggests that mammalian photoreception could be far more complex than previously understood. Furthermore, the mechanisms by which they transduce photic signals to control physiology, or are affected in pathophysiological conditions, are either poorly understood or unknown. Thus, it remains to be shown that physiological levels of light can activate Opn3 in the mammalian brain. Mental health disorders, such as depression, seasonal affective disorder (SAD; via a Pro10Leu mutation in a related opsin gene, *OPN4*; Roecklein et al., 2009), anxiety, schizophrenia, bipolar disorder, Alzheimer’s disease, and other forms of dementia are associated with photosensory disruption (Foster et al., 2013). Moreover, there is a season-of-conception/birth dependent risk to develop these disorders (Foster and Roenneberg, 2008), which further indicates that light may play a crucial role for establishing optimal neural connections. Recently, studies have begun to uncover the neural basis linking light detection and depression/elation. One such study identified a suprachiasmatic nucleus-independent pathway, where photosensitive RGCs project to the perihabenular region of the thalamus, a retina-brain circuit that is strongly implicated in modulating mood (Fernandez et al., 2018). Given the detection of *Opn3*-eGFP in both the retina and thalamus, it is possible that dysfunction of Opn3-dependent photosensory systems may also be involved in mood regulation. What functional disturbances a modulation of Opn3 might cause in regions of the developing embryo, as well as adults, remains to be determined. Nonetheless, the temporal and spatial control of widespread, yet distinct, *Opn3* expression raises the possibility that this photopigment and/or other components of the Opn3-dependent phototransduction cascade play important roles in critical neuronal circuits. In conclusion, this study presents a crucial blueprint from which to investigate autonomic and cognitive opsin-dependent neural development and resultant behaviors under physiological and pathophysiological conditions.

## Synthesis

Reviewing Editor: Lynda Erskine, University of Aberdeen Decisions are customarily a result of the Reviewing Editor and the peer reviewers coming together and discussing their recommendations until a consensus is reached. When revisions are invited, a fact-based synthesis statement explaining their decision and outlining what is needed to prepare a revision will be listed below. The following reviewer(s) agreed to reveal their identity: Yu-Chieh Chen.

Thank you for submitting your revised manuscript. As you will see from the comments below both reviewers are agreed that the manuscript is improved substantially. One issue that remains is the variability of Opn3-eGFP signals. This should be addressed by reporting the number of independent samples and commenting on the variability observed (as detailed below).

Synthesis of the reviewers’ comments:

The authors have addressed the majority of the reviewers’ comments. While small concerns remain, the data presented in the manuscript would be a useful resource for developmental biologists and for understanding OPN3 expression and function.

The added cartoon schemes and the revised table 1 have greatly improved the readability of the manuscript. The redundancy of the result and discussion sections in the previous manuscript has been removed. I would like to thank the authors for the detailed explanation for the technical problems regarding the co-staining with glial markers in early embryonic stages and appreciate the added experiments at E17.5 with GFAP co-staining (extended fig. 7-1).

The only point that the authors have not appropriately addressed is the quantification of the Opn3-eGFP expression. The authors stated that “Moreover, proper quantitative analyses of the expression levels of Opn3 using western blotting would require a trust-worthy Opn3 antibody, which currently is not commercially or academically available.” However, the concern is not about quantifying the expression level of endogenous Opn3, but instead about the degree of variability of Opn3-eGFP signals among the same and/or across different tissues. It is easy to state that these are “representative” images, but without appropriate data quantifications, the rigor of the data is still missing. Also, the authors argued that the quantification is not the scope of the manuscript. We disagree with this view since the readers should have a way to access whether the expression of Opn3-eGFP in certain regions of interest is consistently observed by themselves instead of looking up the description in Table 1. At the very least, the authors should state how many samples were collected in each “representative” image and point out any variability observed so that future researchers have some basis to refer to when they observe any discrepancy in Opn3-eGFP expression.

Minor: In lines 151 and 156, the authors missed the references for the antibodies used for GFAP co-staining experiments.

## Author Response

Dear Lynda,

We thank you for sending us the reviewer’s comments on our manuscript eN-NWR-0141-21R1, and for your accompanying editorial letter. We are pleased that the reviewers agreed that our manuscript has improved substantially and that you all find our manuscript potential suitable for publication in eNeuro.

Below, we have addressed the minor comments and have revised our manuscript accordingly. Line references refers to the Clean Copy of the manuscript.

Synthesis Statement for Author (Required):

Thank you for submitting your revised manuscript. As you will see from the comments below both reviewers are agreed that the manuscript is improved substantially. One issue that remains is the variability of Opn3-eGFP signals. This should be addressed by reporting the number of independent samples and commenting on the variability observed (as detailed below).

Synthesis of the reviewers’ comments:

The authors have addressed the majority of the reviewers’ comments. While small concerns remain, the data presented in the manuscript would be a useful resource for developmental biologists and for understanding OPN3 expression and function.

The added cartoon schemes and the revised table 1 have greatly improved the readability of the manuscript. The redundancy of the result and discussion sections in the previous manuscript has been removed. I would like to thank the authors for the detailed explanation for the technical problems regarding the co-staining with glial markers in early embryonic

stages and appreciate the added experiments at E17.5 with GFAP co-staining (extended fig. 7-

1).

1. The only point that the authors have not appropriately addressed is the quantification of the

Opn3-eGFP expression. The authors stated that “Moreover, proper quantitative analyses of the expression levels of Opn3 using western blotting would require a trust-worthy Opn3 antibody, which currently is not commercially or academically available.” However, the concern is not about quantifying the expression level of endogenous Opn3, but instead about the degree of variability of Opn3-eGFP signals among the same and/or across different tissues. It is easy to state that these are “representative” images, but without appropriate data quantifications, the rigor of the data is still missing. Also, the authors argued that the

quantification is not the scope of the manuscript. We disagree with this view since the readers should have a way to access whether the expression of Opn3-eGFP in certain regions of interest is consistently observed by themselves instead of looking up the description in Table

1. At the very least, the authors should state how many samples were collected in each “representative” image and point out any variability observed so that future researchers have some basis to refer to when they observe any discrepancy in Opn3-eGFP expression.

We appreciate the above clarification regarding “the quantification of the Opn3-eGFP expression” matter, which we agree is relevant information. In this manuscript, the presented results of Opn3-eGFP expression patterns are consistent observations with no detectable variations comprised from analyzing a minimum of three and up to five specimens of each time

point. This is now clarified both in the material and methods (lines 132-133), and the result section (lines 193-195).

2. Minor: In lines 151 and 156, the authors missed the references for the antibodies used for

GFAP co-staining experiments.

To facilitate the double-blind review process the above mentioned references were deliberately not included in the manuscript. The references are now included in the manuscript.

With these additional minor clarifications we hope that you will find our manuscript suitable for publication in eNeuro.
