# Supplementary figures and images for: Distinct Opsin 3 (Opn3) Expression in the Developing Nervous System during Mammalian Embryogenesis
Source: eNeuro. 2021 Sep 9;8(5):ENEURO.0141-21.2021. doi: 10.1523/ENEURO.0141-21.2021 (PMC8445036; doi:10.1523/ENEURO.0141-21.2021)

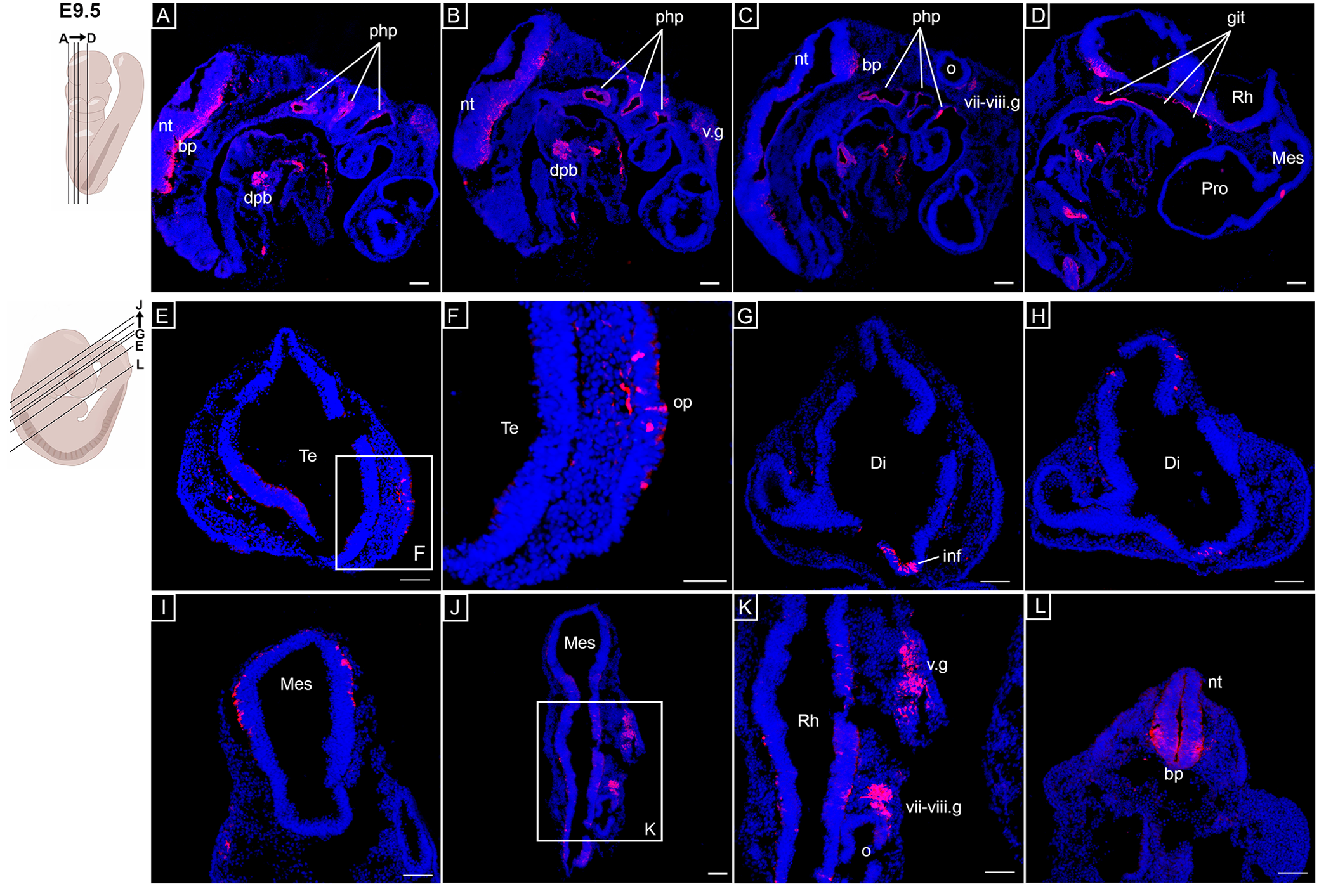

Supplement: Extended Data Figure 1-1 — Opn3-eGFP immunodetection (red) at E9.5 in sagittal (A–D) and coronal (E–L) serial sections counterstained with DAPI (blue). On the left, schematics of E9.5 embryos including planes of sections shown in A–L. A, bp: basal plate, dpb: dorsal pancreatic bud, nt: neural tube, php: pharyngeal pouches. B, dpb: dorsal pancreatic bud, nt: neural tube, php: pharyngeal pouches, v.g: trigeminal ganglion. C, bp: basal plate, nt: neural tube, o: otic vesicle, php: pharyngeal pouches, vii-viii.g: facio-acoustic ganglia. D, git: gastrointestinal tract, Mes: mesencephalic vesicle, Pro: prosencephalic vesicle, Rh: rhombencephalic vesicle. E, Te: telencephalic vesicle. F, op: olfactory placode, Te: telencephalic vesicle. G, Di: diencephalic vesicle, inf: infundibulum. H, Di: diencephalic vesicle. I, Mes: mesencephalic vesicle. J, Mes: mesencephalic vesicle. K, o: otic vesicle, Rh: rhombencephalic vesicle, v.g: trigeminal ganglion, vii-viii.g: facio-acoustic ganglia. L, bp: basal plate, nt: neural tube. Scale bars: 100 μm. Download Figure 1-1, TIF file. [file enu-eN-NWR-0141-21-s05.tif]

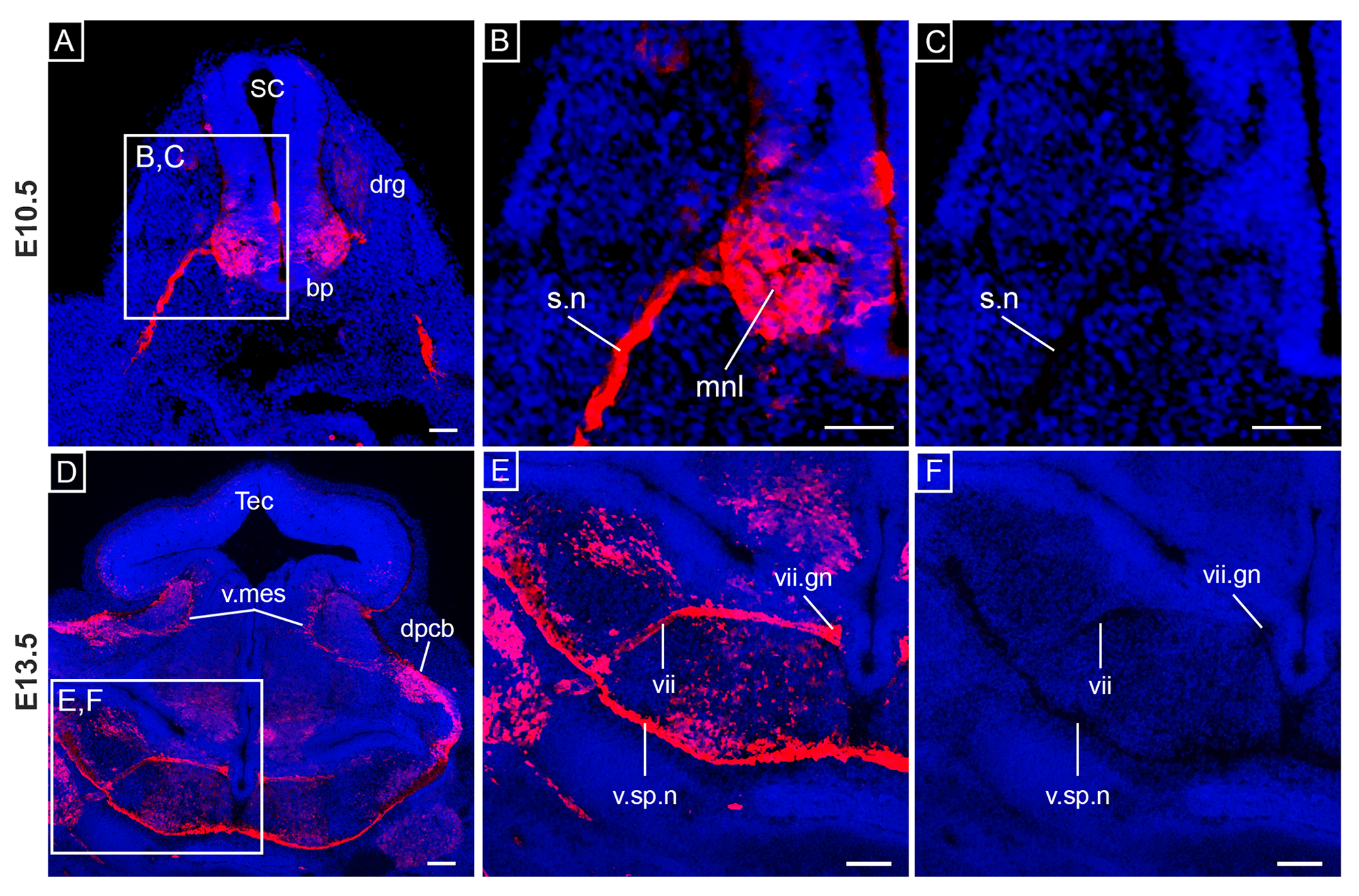

Supplement: Extended Data Figure 1-2 — No DAPI labelled nuclei were detected in Opn3-eGFP-positive projection areas. A–F, Opn3-eGFP immunodetection (red) at E10.5 in (A–C) and at E13.5 (D–F) horizontal sections counterstained with DAPI (blue). A, bp: basal plate, drg: dorsal root ganglion, SC: spinal cord. B, s.n: spinal nerves, mnl: mantle layer. C, s.n: spinal nerves. D, dpcb: deep nuclei of cerebellum, Tec: tectum of midbrain, v.mes: mesencephalic trigeminal nucleus. E, v.spn: spinal trigeminal nerve, vii: facial nerve, vii.gn: genu of facial nerve. F, v.spn: spinal trigeminal nerve, vii: facial nerve, vii.gn: genu of facial nerve. Scale bars: 100 μm. Download Figure 1-2, TIF file. [file enu-eN-NWR-0141-21-s06.tif]

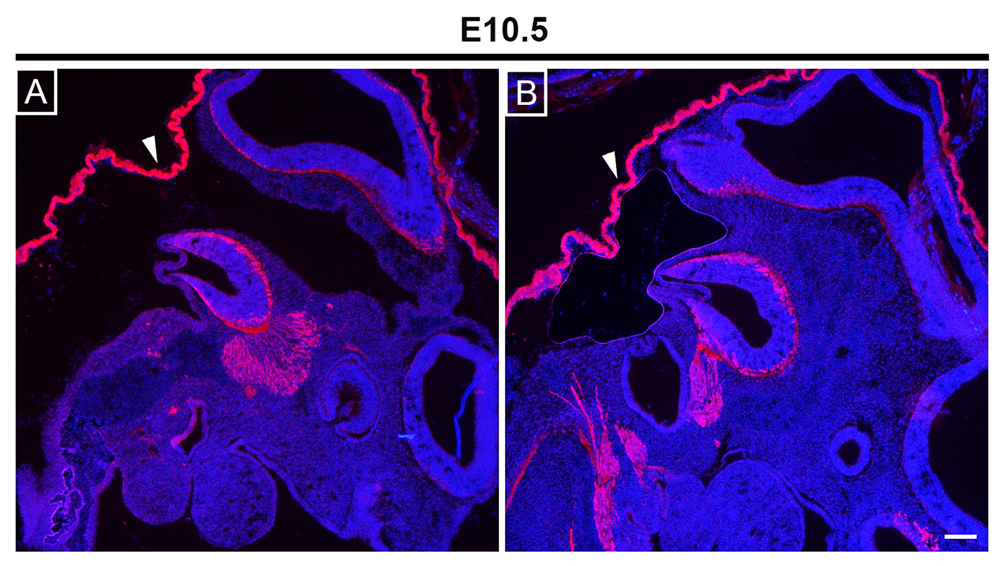

Supplement: Extended Data Figure 1-3 — Raw data of the main images in Figure 1A,B. Opn3-eGFP immunodetection (red) at E10.5 in horizontal serial sections counterstained with DAPI (blue) showing the (in Fig. 1A,B) digitally removed Opn3-eGFP-positive amnion surrounding the embryo (arrowheads). Scale bar: 100 μm. Download Figure 1-3, TIF file. [file enu-eN-NWR-0141-21-s07.tif]

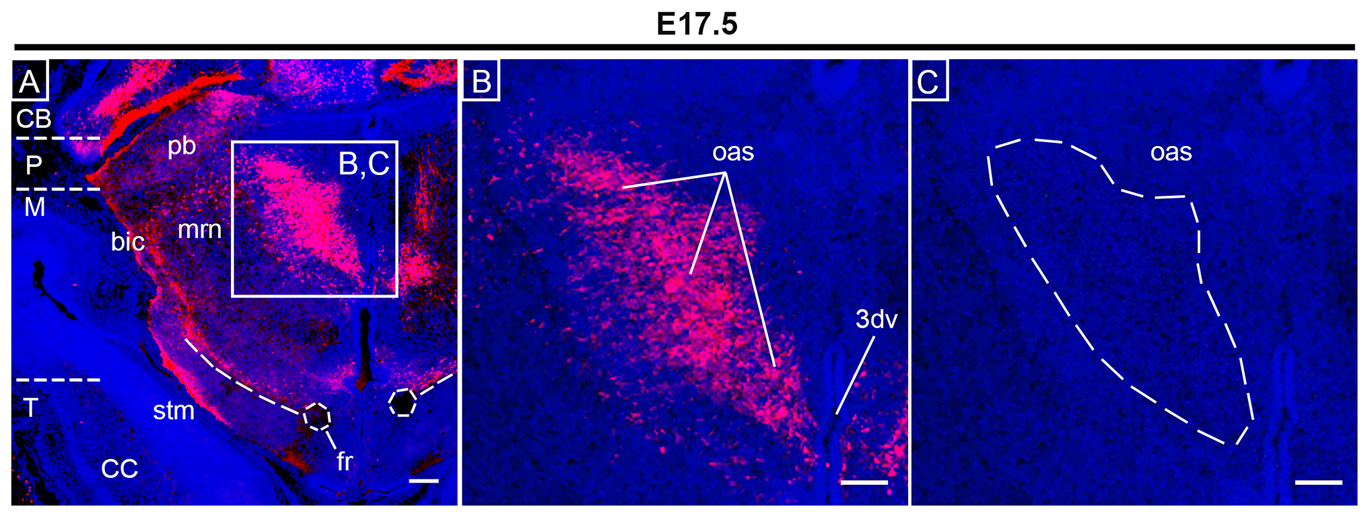

Supplement: Extended Data Figure 6-1 — Confirmation of DAPI labelled nuclei in the region of the oculomotor associated subnuclei. A–C, Opn3-eGFP immunodetection (red) at E15.5 in horizontal (A–C) sections counterstained with DAPI (blue). A, bic: brachium of inferior colliculus, CB: cerebellum, CC: cerebral cortex, fr: fasciculus retroflexus, M: midbrain, mrn: midbrain reticular nuclei, P: pons, pb: parabrachial nuclei, stm: stria medullaris, T: thalamus. B, oas: oculomotor associated subnuclei. C, 3dv: third ventricle. Scale bars: 100 μm. Download Figure 6-1, TIF file. [file enu-eN-NWR-0141-21-s08.tif]

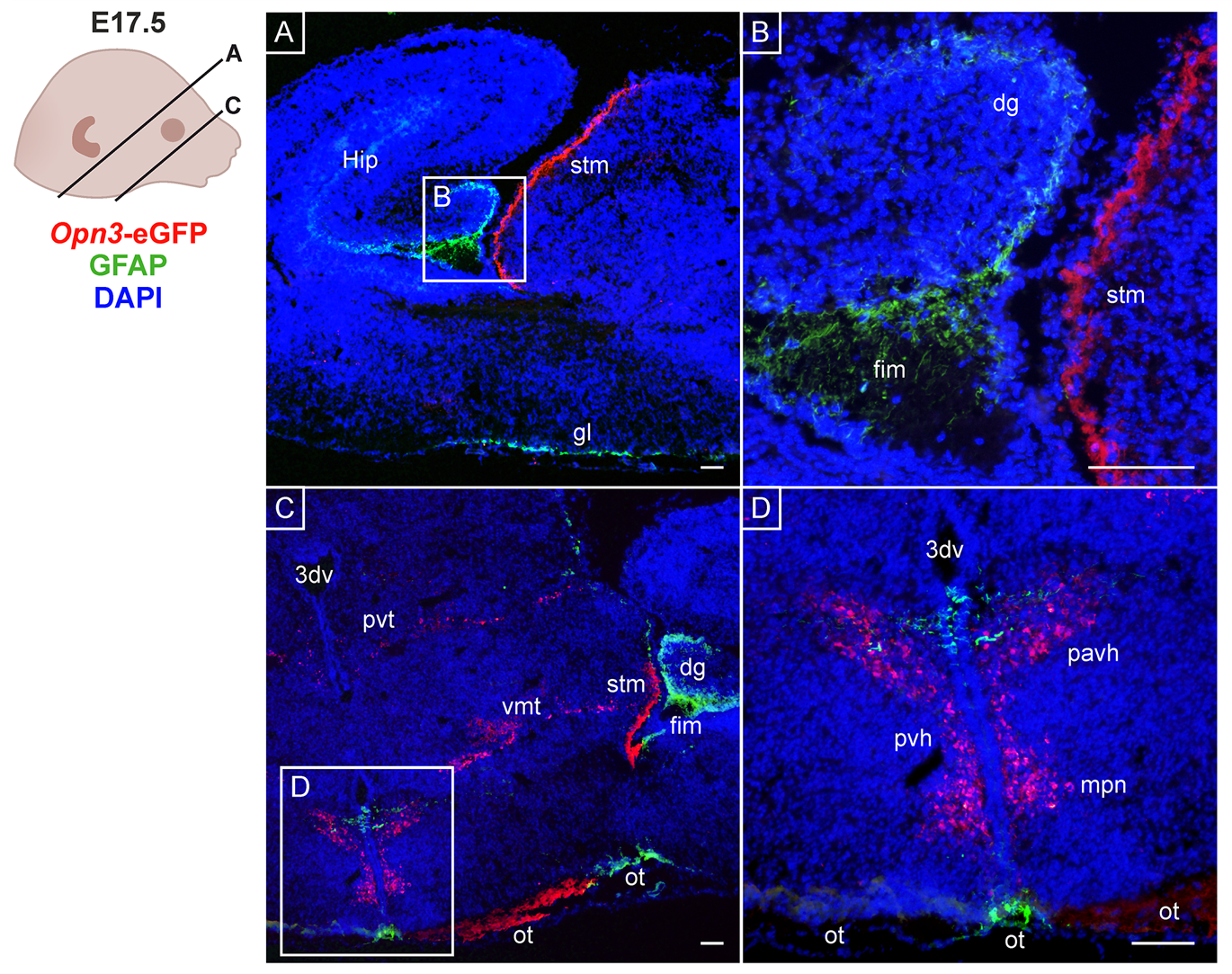

Supplement: Extended Data Figure 7-1 — Opn3-eGFP expression was not observed in GFAP+ astrocytes. On the left, a schematic of an E17.5 head including planes of sections shown in A–D. A–D, Opn3-eGFP (red) and GFAP (green) immunodetection at E17.5 in coronal sections counterstained with DAPI (blue). Opn3-eGFP and GFAP was not co-expressed in GFAP+ astrocytes in the hippocampal, thalamic or hypothalamic regions. C, D, Minimal co-localization, but not co-expression, of Opn3-eGFP and GFAP was observed in parts of the optic tract. A, gl: glia limitans, Hip: hippocampus, stm: stria medullaris. B, dg: dentate gyrus, fim: fimbria, stm: stria medullaris. C, 3dv: third ventricle, dg: dentate gyrus, fim: fimbria, ot: optic tract, pvt: paraventricular thalamic nucleus, stm: stria medullaris, vmt: ventromedial thalamic nucleus. D, 3dv: third ventricle, mpn: medial preoptic nucleus, ot: optic tract, pavh: paraventricular hypothalamic nucleus, pvh periventricular hypothalamic nucleus. Scale bars: 100 μm. Download Figure 7-1, TIF file. [file enu-eN-NWR-0141-21-s09.tif]

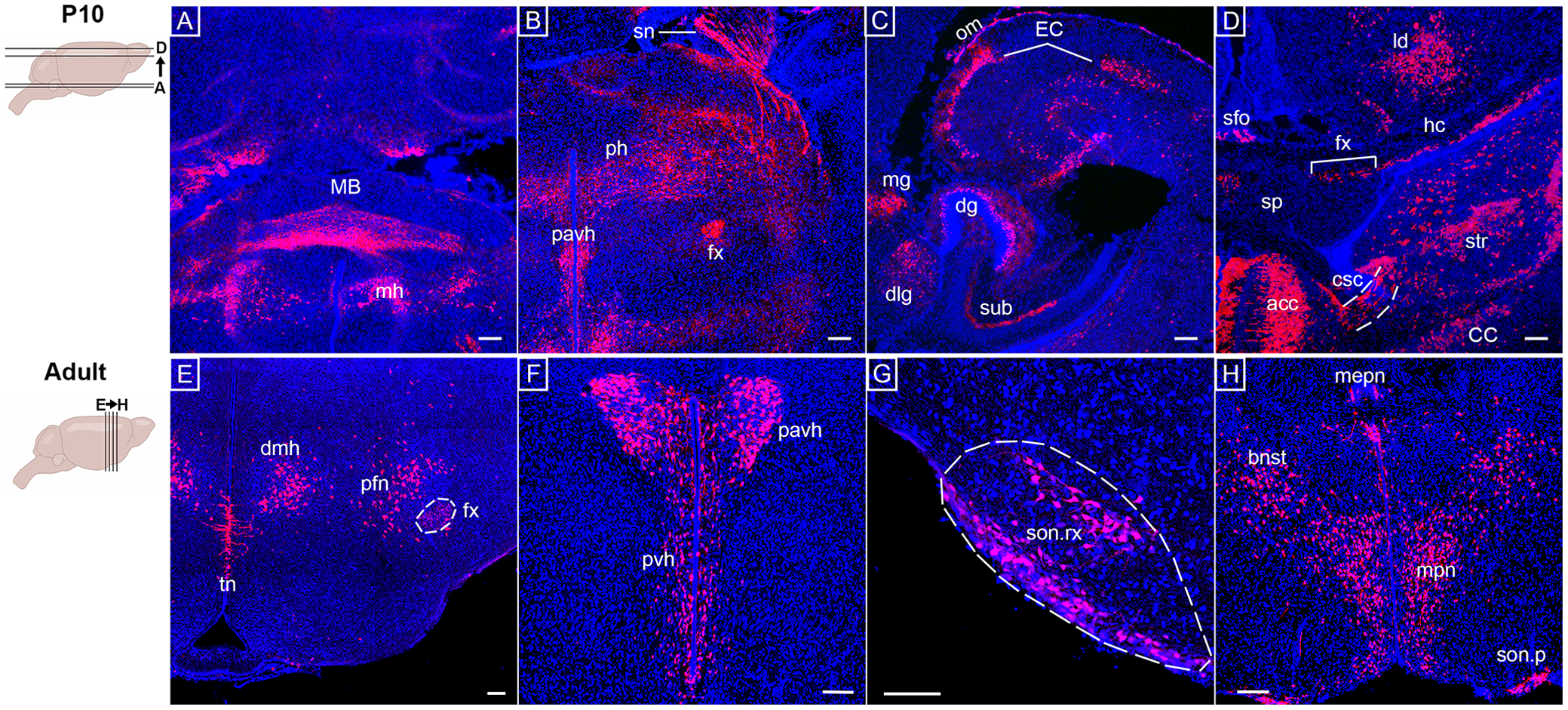

Supplement: Extended Data Figure 8-1 — Opn3-eGFP immunodetection (red) at P10 in horizontal (A–D) and at adult stage in coronal (E–H) serial sections of forebrain areas counterstained with DAPI (blue). On the left, schematics of P10 and adult brains including planes of sections shown in A-H. A, MB: mammillary bodies, mh: medial hypothalamic nuclei. B, fx: posterior fibers of the fornix, pavh: paraventricular hypothalamic nucleus, ph: posterior hypothalamus, sn: substantia nigra. C, dg: dentate gyrus, dlg: dorsal lateral geniculate nucleus, EC: entorhinal cortex, mg: medial geniculate nucleus, om: outer membrane, sub: subiculum. D, acc: anterior cingulate cortex, CC: cerebral cortex, fx: fibers of fornix, hc: hippocampal commissure, ld: lateral dorsal thalamic nucleus, sfo: subfornical organ, str: striatum, sp: septal nuclei. E-H, Opn3-eGFP immunodetection using an anti-rabbit 488 antibody, here pseudo-colored (from green to red). E, dmh: dorsomedial hypothalamic nucleus, fx: fibers of the fornix, pfn: perifornical lateral hypothalamic nuclei, tn: tanycytes. F, pavh: paraventricular hypothalamic nucleus, pvh: periventricular hypothalamic nucleus. G, son.rx: retrochiasmatic supraoptic nucleus. H, bnst: bed nucleus of stria terminalis, mepn: median preoptic nucleus, mpn: medial preoptic nucleus, son.p: supraoptic nucleus proper. Scale bars: 100 μm. Download Figure 8-1, TIF file. [file enu-eN-NWR-0141-21-s10.tif]
